# Incorporating the possibility of cure into network meta-analyses: A case study from resected Stage III/IV melanoma

**DOI:** 10.1017/rsm.2025.10038

**Published:** 2025-10-15

**Authors:** Keith Chan, Sarah Goring, Kabirraaj Toor, Murat Kurt, Andriy Moshyk, Jeroen Jansen

**Affiliations:** 1 Precision AQ, Vancouver, BC, Canada; 2 Bristol Myers Squibb, Princeton, NJ, USA; 3 School of Pharmacy, University of California San Francisco, San Francisco, CA, USA

**Keywords:** fractional polynomial, melanoma, mixture cure model, network meta-analysis

## Abstract

In many areas of oncology, cancer drugs are now associated with long-term survivorship and mixture cure models (MCM) are increasingly being used for survival analysis. The objective of this article was to propose a methodology for conducting network meta-analysis (NMA) of MCM. This method was illustrated through a case study evaluating recurrence-free survival (RFS) with adjuvant therapy for stage III/IV resected melanoma. For the case study, the MCM NMA was conducted by: (1) fitting MCMs to each trial included within the network of evidence; and (2) incorporating the parameters of the MCMs into a multivariate NMA. Outputs included relative effect estimates for the MCM NMA as well as absolute estimates of survival (RFS), modeled within the Bayesian multivariate NMA, by incorporating absolute baseline effects of the reference treatment. The case study was intended for illustrative purposes of the MCM NMA methodology and is not meant for clinical interpretation. The case study demonstrated the feasibility of conducting an MCM NMA and highlighted key issues and considerations when conducting such analyses, including plausibility of cure, maturity of data, process for model selection, and the presentation and interpretation of results. MCM NMA provides a method of comparative survival that acknowledges the benefit newer treatments may confer on a subset of patients, resulting in long-term survival and reflection of this survival in extrapolation. In the future, this method may provide an additional metric to compare treatments that is of value to patients.

## Highlights

### What is already known?

- Current methods are available to include sophisticated survival models such as piecewise, spline, fractional polynomial, mixture, and cure models
- Additionally, indirect treatment comparison methods such as network meta-analysis (NMA) are employed to obtain relative treatment effects between treatments; however, applications of the NMA framework to MCMs are limited
- The objective of this article is to propose a methodology for conducting an MCM NMA, illustrated with an example case study in melanoma

### What is new?

- Details for a method incorporating MCM within two-step NMA is outlined in this article with an example in melanoma.

### Potential impact for RSM readers

- Readers of RSM can use this article to explore additional types of survival modelling and contribute/comment on the development of these methods.

## 1 Introduction

Cost-effectiveness analysis (CEA) of interventions that aim to improve survival in oncology is typically performed for a lifetime horizon. However, it is often the case that available data from clinical trials informing the CEA may have limited follow-up, thereby requiring extrapolation of overall survival (OS), progression-free survival (PFS), or recurrence-free survival (RFS) estimates for the compared interventions.

For fitting survival models, initial recommendations from the National Institute for Health and Care Excellence Decision Support Unit (NICE DSU) were to fit standard parametric models.^1^ However, recommendations were recently updated to include more sophisticated survival models such as piecewise, spline, fractional polynomial, mixture, and cure models.^1^ ^,^ ^2^ These latter models have greater flexibility for capturing complex hazard functions such as those associated with newer immunotherapies used in oncology, which typically involve a delayed response to treatment and long-term survival or cure for a subset of patients.^3^ ^–^ ^6^ With many of these modeling approaches, extrapolations are unconstrained and require assessment of clinical plausibility among models that fit well to the observed data; however, with certain cure models, the survival function will necessarily plateau.

With mixture cure models (MCM), a proportion of the treated population is explicitly modeled as being “cured”—that is, the risk of death comes only from background mortality rates aligned with an age- and sex-matched general population; the remaining proportion captures the excess mortality associated with the disease.^7^ ^–^ ^10^ With the increasing use of novel interventions potentially providing a cure for a subset of individuals, MCMs may be considered to explore underlying survival heterogeneity and to extrapolate survival data characterized by “flattening tails.” This is particularly relevant in oncology, where long-term survival (often interpreted as 5-year survival), indicative of the “flattening tails” in the observed survival, is becoming more common.^11^ ^,^ ^12^

Although the existing guidance from the NICE DSU focuses on single-trial settings, comparative data between an intervention and all relevant comparators of interest are needed for health technology assessments. Often, indirect treatment comparison methods such as network meta-analysis (NMA) are employed to obtain relative treatment effects between treatments. And while methods exist for NMAs incorporating survival models such as parametric distributions,^13^ ^,^ ^14^ and fractional polynomial functions,^15^ ^,^ ^16^ applications of the NMA framework to MCMs are limited.^17^ ^–^ ^23^

The objective of this article is to propose a methodology for conducting an MCM NMA. The implementation of this method is illustrated with an example case study in melanoma. The article is organized as follows: first, we revisit MCM and present the MCM NMA framework; next, we present the methods and results of the case study; finally, we conclude with a discussion of the key issues and considerations when conducting MCM NMA.

## 2 Developing and applying mixture cure modeling in the context of network meta-analysis

### 2.1 Mixture cure models

Since their introduction about 75 years ago by Boag et al.^24^ and Berkson and Gage,^7^ MCMs have been widely studied and further developed as a statistical tool for survival analysis.^25^ Under the premise that cured patients will never experience an “event,” cure models were initially defined as:
(1)

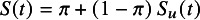

 where *S(t)* is the probability of no event at time *t* for the overall population, 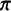 is the proportion of patients that are considered cured (that is the cure fraction), and *S_u_(t)* is the probability of no event at time *t* for the subset of patients who are uncured.^26^ However, as patients may die from other causes, the model has since been extended to incorporate background mortality:
(2)

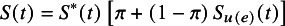

 where S*(t) is the background mortality for the population of interest and *S_u(e)_(t)* represents the excess mortality among the uncured. In Equation 2, all-cause mortality among the cured is captured by S*(t) while all-cause mortality among the uncured is obtained by multiplying the survival functions S*(t) and *S_u(e)_(t)*. This approach is equivalent to adding the associated hazards and is valid as long as hazard rates are independent.^27^

In the context of randomized clinical trials (RCTs), independent MCMs may be fit to each arm of the trial.^6^ ^,^ ^28^ Alternatively, both the cure fraction, 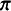 , and uncured survival function, *S_u(e)_(t)*, in Equation 2 can be parameterized using covariates, allowing treatment effects to be incorporated into the MCM using treatment arm as a covariate.

For the cure fraction, 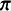 , the logit transformation is typically applied, with covariate effects expressed as log odds ratios. Alternative link functions have been explored, although are less commonly used.^25^ Background mortality, S*(t), is presumed to be the same across treatment arms, however, treatment effects may also be applied to *S_u(e)_(t)*, with the approach depending on the parametric distribution. The distributions considered for *S_u(e)_(t)* in the current article include exponential, Weibull, log-logistic, and log-normal. Of these four distributions, the exponential is characterized by one parameter and thus, can only involve one treatment effect parameter. The other three distributions are characterized by two parameters, allowing treatment effects to be applied to one or both parameters. By using the logit transformation for the cure fraction and transformations for *S_u_(t)* (detailed in Supplementary Appendix A), parameters can follow a multivariate normal distribution with accompanying covariance matrix.

### 2.2 Network meta-analysis models

When adopting a meta-analysis framework, analysis is traditionally performed on the relative treatment effect scale.^29^ ^,^ ^30^ Hence, the meta-analysis of MCMs focuses on treatment effects on both the cure fraction, 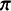 , and uncured survival function, *S_u(e)_(t).* In this section, we first describe standard univariate NMA models that are used to analyze a single treatment effect parameter. Next, we describe multivariate NMA models, which can simultaneously synthesize multiple parameters, making them suitable for synthesizing the multiple parameters of the MCM.

#### 2.2.1 Univariate network meta-analysis

When the available evidence consists of a network of multiple pairwise comparisons (e.g., AB-trials, AC-trials, BC-trials, etc.), the standard univariate random effects model for NMA can be specified as follows^30^:
(3)

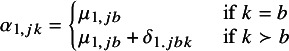



There are *k* treatments labeled as A, B, C, etc., and treatment A is taken to be the reference treatment for the analysis. 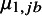 is the (transformed) outcome in study *j* on “baseline” treatment *b*, which will vary across studies. 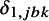 is the trial-specific treatment effect of *k* relative to treatment *b*. These trial-specific effects are drawn from a random-effects distribution: 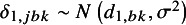 . The pooled effects 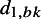 are identified by expressing them in terms of the reference treatment A: 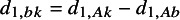 , with 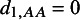 . The heterogeneity 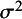 is assumed constant for all treatment comparisons (a fixed effect model is obtained if 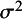 equals zero).

#### 2.2.2 Multivariate network meta-analysis

Univariate NMA models were extended by Achana et al. to simultaneously synthesize treatment effects related to multiple outcomes, and later adapted by Cope et al. to synthesize multiple parameters of a survival function^31^ ^,^ ^32^:

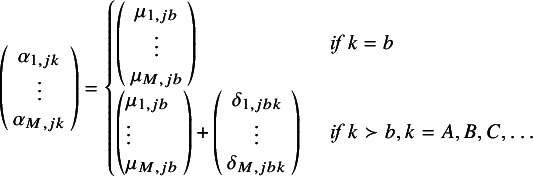



(4)

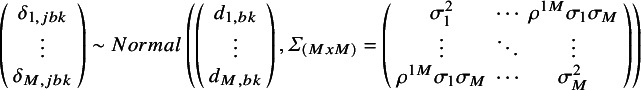




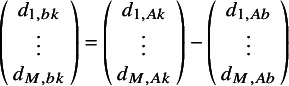




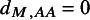

where 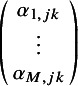 are the *M* expected (transformed) outcomes 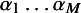 for treatment *k* in trial *j*. 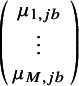 are the expected (transformed) outcomes in study *j* with “baseline” treatment *b*. 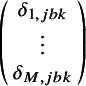 are the trial-specific treatment effect of *k* relative to treatment *b*, which are drawn from a multivariate normal distribution with mean relative treatment effects 
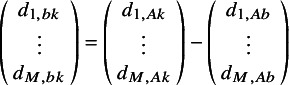
 and the corresponding covariance matrix 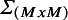 . Under a fixed effect model, the multivariate normal distribution with the pooled estimates would be replaced with 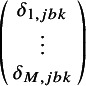 = 
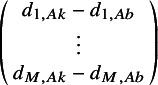
 , and consequently, the between-study covariance matrix would not need to be estimated.

### 2.3 Multivariate network meta-analysis of mixture cure model

In this article, we introduce the use of a multivariate NMA framework to simultaneously model the parameters of an MCM, as follows:
(5)

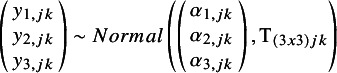

 where 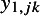 represents the log odds of the cure fraction with treatment *k* in study *j*; 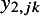 and 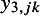 are the transformed parameters of the uncured survival distribution, *S_u(e)_(t)*, (with transformations as defined in Appendix A); and 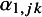 , 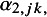 and 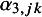 are the corresponding expected underlying estimates. 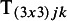 is the corresponding covariance matrix with treatment *k* in study *j*.

Consistent with the application of multivariate NMA for survival parameters used by Cope et al.,^31^ the MCMs are first fit for the trial-specific time-to-event outcomes of interest to identify the relevant parameters (and their correlations), which are then used as inputs in a multivariate NMA.

## 3 Illustration with a case study

### 3.1 Methods

To apply the MCM NMA methodology outlined in the previous section, a case study was conducted to compare RFS between different adjuvant therapies for resected melanoma using an MCM NMA. The case study is intended for illustrative purposes of the MCM NMA methodology and is not meant for clinical interpretation.

#### 3.1.1 Data identification and preparation

The evidence informing the case study was based on a previously presented systematic review (details in Supplementary Appendix B).^22^ ^,^ ^33^

In the network of evidence, published RFS Kaplan–Meier (KM) curves were digitized and survival proportions over time recorded, together with reported numbers at risk under the survival curve. Based on this information, the algorithm by Guyot et al.,^34^ was used to reconstruct survival and censoring times from the published KM curves to facilitate analyses as if one had individual patient-level data (IPD) for the time-to-event outcomes from these RCTs. These reconstructions were superseded by IPD, where available.

#### 3.1.2 Background mortality

Background mortality was estimated for each trial based on life tables in conjunction with the study population’s age at baseline, sex, and study site location (details in Supplementary Appendix B). For trials for which IPD was available, the probability of each patient dying at any point in time (up to 100 years) was determined based on the life-table information. For trials without available IPD for baseline characteristics, pseudo IPD was simulated using the reported proportion of patients at each study site, mean age with standard deviation, and proportion of males.

Using either true IPD or pseudo IPD, corresponding mortality data were generated based on life tables (country-specific World Health Organization [WHO] life tables). Survival distributions (exponential, log-logistic, log-normal, Weibull, and Gompertz) were then fit to these trial-specific background mortality data. The best-fitting distribution according to AIC was used to represent the trial-specific background mortality function, ensuring that the background mortality used to generate cure models for each trial aligned with the source population of that trial. As the trials were relatively similar in terms of age, sex, and geographic distributions (details in Supplementary Appendix B), the estimated trial-specific background mortality was also relatively similar (details in Supplementary Appendix D).

For a given trial arm, background mortality was incorporated into the cure model as expected hazards based on the trial’s best-fitting background mortality survival distribution at the time of each death or censoring event. These expected hazards were input into the model as a vector of length equal to the sample size of the trial arm, and provided the benchmark against which excess hazards could be estimated.

#### 3.1.3 Trial arm mixture cure model fit and selection

Using the trial-specific time-to-event data and background mortality as inputs, MCMs were fit to each trial arm. The uncured survival distributions considered were exponential, Weibull, log-logistic, and log-normal. For each trial arm, model fit was assessed based on the Akaike information criterion (AIC). As the AIC gives more weight to model fit at the start of follow-up (where data density is greatest), the assessment of model fit was further supplemented by clinical plausibility of the model fit and projections near the end of follow-up, including visual inspection of the tails, and clinical plausibility of the estimated cure fraction. This was performed for each trial arm, as well as across arms of each trial.

To conduct the NMA, the current implementation requires that parameterization of the uncured survival distribution, *S_u(e)_(t)*, is the same across all trial arms; hence, a single parameterization for the full network of evidence was selected. This choice was made using the aggregate AIC, calculated across trial arms, balanced against the trial-arm specific evaluations, to ensure that the selected model parameterization was both well-fit according to the AIC and clinically plausible across all trial arms. Additionally, goodness of fit was assessed using residuals (details in Supplementary Appendix E). Using the selected parameterization of uncured survival distribution, arm-specific transformed estimates for the cure fractions and uncured survival function parameters, along with the covariance matrices, were obtained for all trial arms.

#### 3.1.4 Bayesian multivariate network meta-analysis

These arm-specific transformed estimates were used as input into a Bayesian multivariate NMA (see Equation 5) using non-informative prior distributions for its parameters. Relative treatment effects regarding the transformed cure fraction and uncured survival parameters with each treatment relative to a chosen reference treatment were estimated using a Markov Chain Monte Carlo (MCMC) method.^35^ Relative treatment effect estimates for the cure fraction were expressed as odds ratios (median of the posterior distribution) along with 95% credible intervals (95% CrI, based on the 2.5th and 97.5th percentile of the posterior distribution). Relative treatment effect estimates for the uncured survival function parameters were expressed as differences between treatments.

In the MCM NMA, relative effect estimates of the cure fraction odds ratios do not necessarily have the same direction of effect as the uncured survival function; a particular treatment can be associated with a higher cure fraction yet worse survival among the uncured, relative to another treatment. Furthermore, when incorporating time-varying hazard ratios, the relative effect among the uncured may change over time, further complicating interpretation. Therefore, to facilitate interpretation and application of the findings to future target populations, the relative treatment effects in terms of cure fractions and survival function parameters were translated into absolute estimates of survival over time by treatment, for the full (combined cured and uncured) population.

These absolute estimates of survival (RFS) were modeled within the Bayesian multivariate NMA model. Pooled estimates were obtained for the overall reference treatment regarding the log odds of the cure fraction and the transformed survival function parameters. Next, the relative effect estimates from the multivariate NMA model were applied to these absolute baseline effects with the reference treatment to obtain estimates for the log odds of the cure fraction and the transformed survival function parameters for all other treatments in the network. For the current case study, there was no pre-specified target population of interest; for illustrative purposes, background mortality was incorporated using the estimate of pooled log hazards of all trials. Based on these treatment-specific absolute estimates and background mortality, the corresponding survival curves were obtained, along with estimates of restricted mean survival time (RMST). These steps were performed as part of the MCMC simulations.

#### 3.1.5 Software and code

The trial-specific MCM analyses were performed with the *flexsurvcure* package in R version 4.1.2 (http://www.r-project.org/). The multivariate NMA was implemented with the JAGS software package (version 4.3.0); model code is provided in Supplementary Appendix C.

### 3.2 Results: Case study, adjuvant therapy for resected melanoma

**Figure 1:**
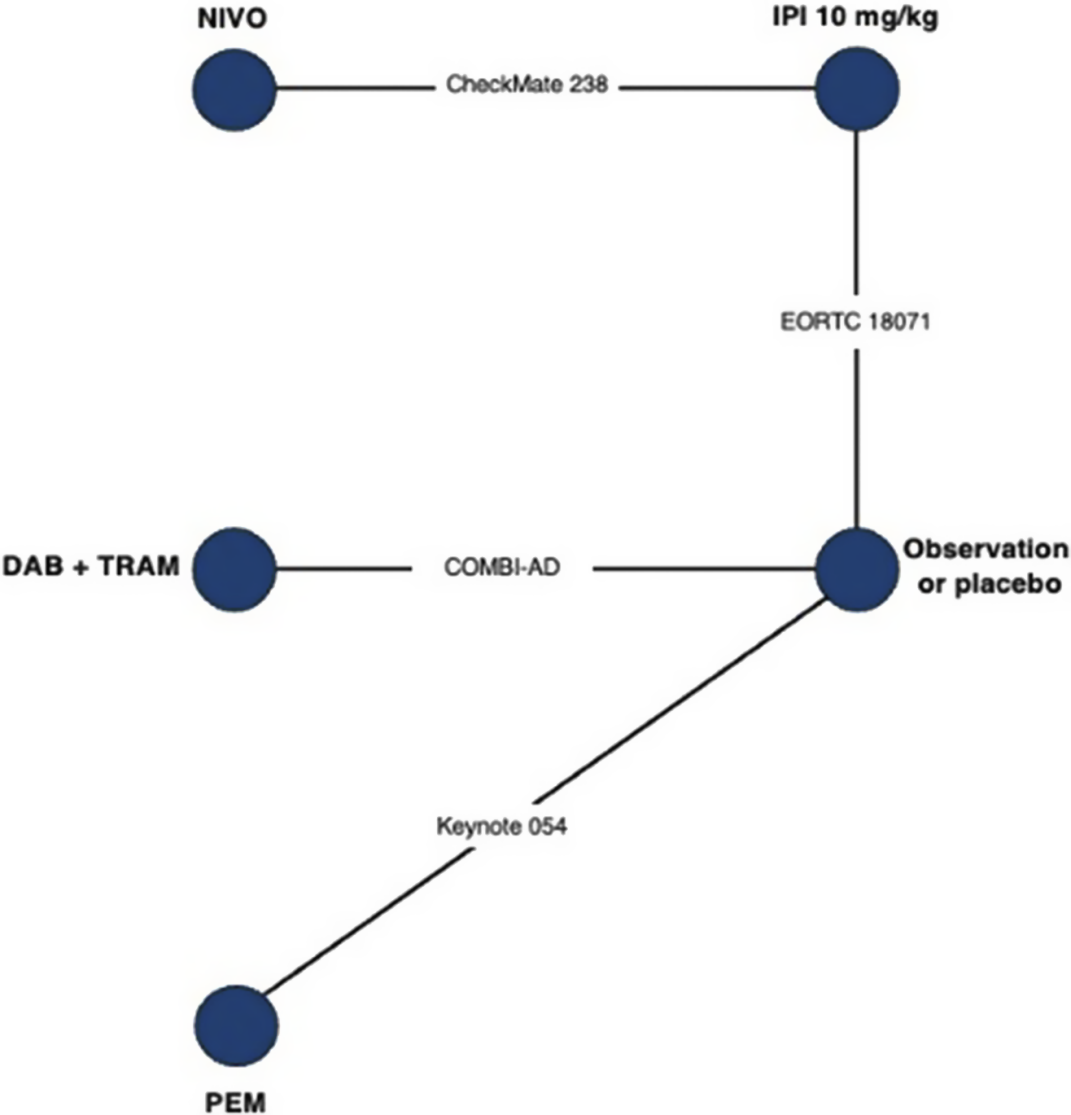
Network of evidence informing the case study of adjuvant therapies for resected melanoma. Abbreviations: DAB, dabrafenib 150 mg BID; IPI, ipilimumab; NIVO, nivolumab 3 mg/kg; PEM, pembrolizumab 200 mg/m^2^ or 10 mg/kg; TRAM, trametinib 2 mg once daily.

The evidence base for the case study involved four RCTs evaluating RFS among patients with resectable melanoma. In total, there were eight trial arms, involving five regimens used in the adjuvant treatment setting (Figure 1). Post-surgical observation/placebo was selected to be the reference treatment in the network. IPD were available from one RCT (CheckMate 238); for the other three RCTs, pseudo IPD were generated using KM curves (for RFS time-to-event data), and summary statistics of baseline patient characteristics (for background mortality).

The Gompertz model provided the best fit for background mortality (Supplementary Appendix D). For the trial-arm MCMs, the log-normal model was the best-fitting in five of eight trial arms according to the aggregate AIC, and was considered clinically plausible for all trial arms. The cure fraction estimates did not vary considerably when fit with the log-normal relative to other well-fitting models. Details on the model fit assessments are provided in Supplementary Appendix E. Data inputs to the Bayesian MCM NMA are provided in Supplementary Appendix F.

The MCM NMA model outputs are provided in Table 1, as differences for each of the MCM parameters relative to observation/placebo (see also Supplementary Data in Supplementary Appendix F). Differences in the direction of treatment effect were evident within the cured and uncured populations in the point estimates for nivolumab 3 mg/kg (NIVO) relative to combination dabrafenib and trametinib (DAB+TRAM) (Supplementary Appendix F). The cure fraction OR point estimate favored NIVO over DAB+TRAM (Supplementary Appendix F), whereas the HR for the survival among the uncured favored DAB+TRAM over NIVO over the first 2 years (Supplementary Appendix F).

**Table 1:** Summary of estimated relative treatment effect parameters from mixture cure model network meta-analysis; adjuvant therapy for resected melanoma

	d1	d2	d3			
Treatment	(variance)	(variance)	(variance)	Cov. 1,2	Cov. 1,3	Cov. 2,3
Placebo	Reference	Reference	Reference	–	–	–
Ipilimumab	0.374 (0.035)	0.231 (0.023)	0.05 (0.009)	−0.013	−0.008	0.007
Dabrafenib + trametinib	0.620 (0.034)	0.996 (0.012)	−0.342 (0.013)	−0.009	−0.009	0.006
Pembrolizumab	0.740 (0.084)	0.130 (0.092)	0.238 (0.020)	−0.070	−0.030	0.034
Nivolumab	0.961 (0.067)	0.261 (0.045)	0.008 (0.021)	−0.025	−0.018	0.016

*Notes*: ds correspond to Equation 4, for the three modeled parameters of the mixture cure model, as defined in Equation 5; d1 represents the treatment effect on cure fraction; d2 and d3 represent the treatment effects on the two parameters of the log-normal fit to uncured survival (see also Supplementary Appendix A). d1 is also presented as an odds ratio in Supplementary Appendix F, Table 7; d2 and d3 are transformed to hazard ratios in Supplementary Appendix F, Figure 6. Estimates were generated from a fixed effect model. Abbreviations: Cov., covariance matrix; d, treatment effect.

**Figure 2:**
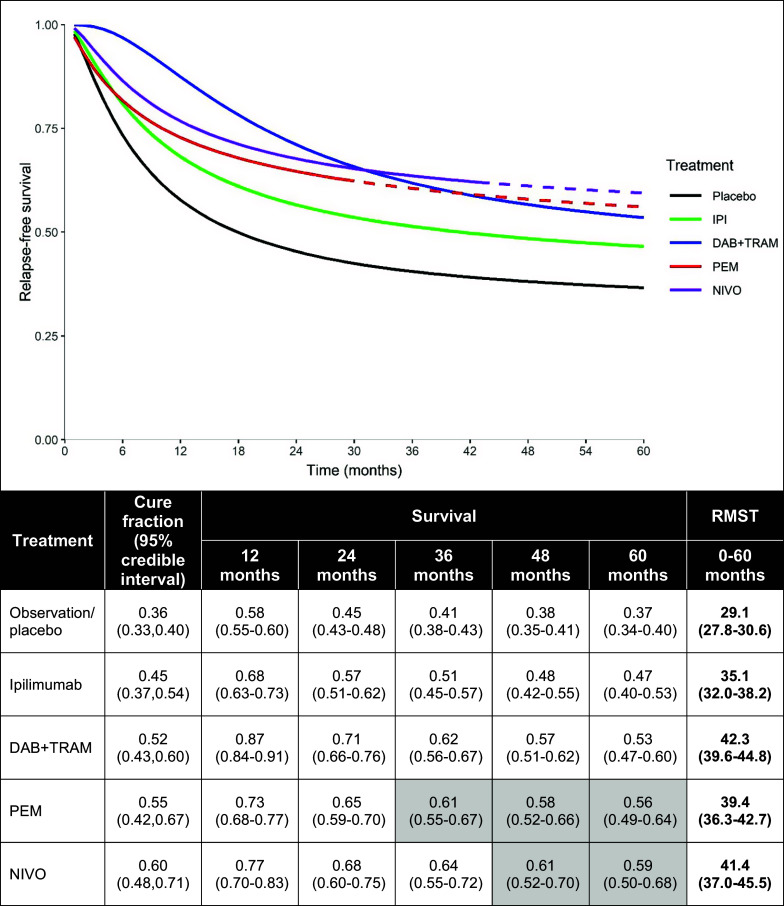
Results for the mixture cure model network meta-analysis; adjuvant therapies for resected melanoma. Notes: Shaded cells represent model extrapolations. Abbreviations: DAB + TRAM, dabrafenib + trametinib; IPI, ipilimumab; NIVO, nivolumab; PEM, pembrolizumab; RMST, restricted mean survival times.

The comparison of treatment-specific absolute estimates of RFS provides a presentation of findings that may be easier to interpret (particularly for non-technical audiences) than the parameter-specific relative effect estimates (Figure 2; with treatment-specific parameter estimates for 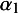 , 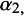 and 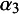 in Table 1). These absolute estimates illustrate the impact of different treatment effects within the cured and uncured groups for the overall (cured + uncured) population. For the comparison between DAB+TRAM relative to NIVO, the initially higher survival among the uncured with DAB+TRAM resulted in initially higher *S(t)* in the combined population, yet the RFS curves crossed at about 30 months, with a higher plateau in the NIVO arm via the higher odds of cure associated with NIVO: approximately 60% of patients (95% CrI: 48%–71%) were estimated as cured in NIVO, compared with approximately 52% (95% CrI: 43%–60%) with DAB+TRAM. In addition to the RFS survival estimates at time *t*, the RMST provides further numerical estimation over the first 60 months, enabling comparison of average RFS times between treatments. Additional plots in Supplementary Appendix E display survival curves separately for the cured and uncured populations, relative to the cure fraction; this further elucidates how the absolute survival rates combine with the cure fraction to produce estimates for the overall population.

## 4 Discussion

In this article, we extended the single-trial analysis framework for MCM to enable multiple comparisons across a network of connected RCTs. The two-step approach we presented in this article, of first fitting MCMs using typical MCM methods^2^ ^,^ ^27^ ^,^ ^36^ and then applying published multivariate NMA models^13^ ^,^ ^32^ for meta-analysis, offers greater transparency and enables wider adoption than developing new, more complex, NMA models specifically for MCMs. This methodological development is valuable because MCM is now a recommended and utilized option for survival modeling within economic evaluations for health technology assessments,^2^ ^,^ ^4^ yet published methods for conducting indirect comparisons using MCMs are sparse.^21^ We anticipate that our current article can help bridge this gap.

The case study presented in this article demonstrated the feasibility of implementing MCM NMA. The MCM NMA model produced clinically plausible treatment-specific estimates of RFS within a “cure” framework, characterized by long-term survivors (i.e., no excess mortality relative to the general population), and evidenced by a plateau in the RFS survival curves. Indeed, the assumption of “cure” for melanoma is considered tenable, and MCMs have previously been implemented within both resectable and unresectable settings.^4^ ^,^ ^6^ ^,^ ^26^ ^,^ ^28^ ^,^ ^36^ ^–^ ^39^

The validity of this assumption—that patients can be “cured” (from a statistical perspective)—is of paramount importance. While MCMs offer a well-established statistical framework for modeling a mixed population of cured and uncured patients, cure models are considered to perform poorly when the cure assumption is flawed and can lead to clinically implausible long-term extrapolations.^2^ Consequently, a key challenge in the application of MCM to a network of RCTs is assessing whether “cure” is valid for all arms of all trials; for example, in disease settings for which newer therapies may offer the potential for cure while older therapies do not. Applying MCM NMAs in other therapeutic settings must be considered carefully, to ensure that there is indeed a fraction of the population who may be cured, and that can be estimated from the available data. If the cure assumption is not tenable, then MCM should not be used; other parametric models may provide a more plausible fit to the data.^13^ ^–^ ^16^ ^,^ ^40^

This relates to another known challenge with MCM, which is estimating cure when data are immature, and when a plateau has not yet emerged within the observed follow-up period.^36^ ^,^ ^41^ ^,^ ^42^ In these situations, Fellizi et al.^36^ suggest using “informed” cure rates, with estimates derived from external sources. Alternatively, such external data could be incorporated into a model selection heuristic,^43^ consistent with guidance issued by the NICE DSU for assessing the validation and justification of the credibility of estimated cure fractions.^2^ Modeling composite endpoints, such as RFS, can be beneficial when OS are immature, provided that the cure assumption is relevant to that endpoint. For these composite survival endpoints the background mortality rate still applies and the background risk of recurrence is set to zero.

Indeed, the model selection process for the current MCM NMA was more complex than for an MCM of an individual trial or trial arm, primarily because the current implementation uses the same choice of survival distribution across the entire network of evidence and a single summary statistic capturing aggregate goodness of fit across the network may be insufficient for identifying a parametric form that is both well-fit to the data and clinically plausible. While this issue is not unique to MCM NMA (other NMAs involving parametric models of hazard functions similarly require a common parametric form, and rely on a multifaceted model selection process), the choice to explicitly estimate the cure fraction in the MCM NMA increases the importance of having clinically plausible extrapolations, and highlights the challenge that the statistical metrics of fit, such as AIC, are largely influenced by the larger sample size at the beginning of the curve, whereas the cure fraction estimates are informed by the tails. As such, a transparent and pre-specified process for model selection is recommended, as well as comprehensive reporting of results for other candidate model forms, as presented in the current case study. Future work on this topic would benefit from increasingly thorough methods of model selection and criteria for validation.

A noteworthy issue in presenting relative treatment effects for MCM NMAs is the difficulty in interpreting the estimated relative treatment effects on their own. While the MCM NMA model outputs capture the odds ratios for cure and the relative survival among the uncured, these populations are not considered separately in decision-making contexts, and cured patients are never known *a priori*. In our case study, we identified a situation where there were opposite directions of effect, such that one treatment was associated with lower odds of cure, yet better survival among the uncured, relative to another. Presented separately, this can be difficult to interpret for the mixed population as a whole. As such, results for the combined survival on the absolute scale (conditional on a baseline model) may be more useful in clinical and decision-making settings, while still enabling comparisons across treatments. As in any NMA application, care should be taken in selecting an appropriate choice of absolute baseline effects.^44^

A key feature of our approach was that we did not require access to IPD for all trials. For time-to-event data, we were able to use KM reconstructions to produce pseudo IPD that was sufficient for fitting MCM models.^34^ However, it remains important with these reconstructions that number-at-risk data are reported and included in the reconstruction, to ensure that censoring has been applied appropriately and to avoid falsely assigning too much weight in the tails and consequently too much certainty around cure fractions.^34^

A limitation of using pseudo IPD rather than true IPD related to covariate adjustment. In Equation 2, we implicitly assumed independence of background and excess mortality; however, this does not hold in the presence of common causal factors, such as age. True IPD would have enabled adjustment of both the excess mortality and the background mortality based on factors such as age, sex, and country of enrolment, whereas in the current model, background mortality was based on pseudo IPD simulated from aggregate-level data according to age, sex, and country of enrolment, and excess mortality was unadjusted. Despite this, the models in our case study appeared sufficient for estimating mortality for each study. Previous research has shown that small differences in background mortality inputs have little impact on parameter estimates^45^; other applications of MCMs have used background mortality rates representing target populations rather than the sampled populations.^3^ ^,^ ^46^ Sensitivity analyses around background mortality inputs may be warranted, especially because MCM implementation involves treating background mortality as a fixed rather than a random variable.

MCM NMA is subject to the same assumptions and limitations as traditional NMA.^47^ For a relevant and valid MCM NMA, each RCT included in the analysis needs to be representative of the target population of interest, which means no differences in effect modifiers regarding the cure fractions and survival for the uncured between each of the study populations and the target population. When there are systematic differences in effect modifiers between the RCTs comparing different subsets of the intervention in the network, the NMA will be biased. Our case study was drawn from previously conducted NMAs,^22^ ^,^ ^33^ in which trials were considered sufficiently similar for generating unbiased estimates of effect via NMA. While it is possible to incorporate baseline covariates into MCMs; however, doing so would require IPD for all trials in the network. The differential impact of effect modifying factors on cure fraction versus uncured survival within an MCM NMA, and methods for adjustment when limited to aggregate-level data, could be explored in future work.

In this article, we illustrated the implementation of MCM NMA using a transparent and accessible method that builds on existing techniques in MCM and NMA modeling. The current work outlined is subject to limitations with regard to MCM as well as the extension to the NMA framework and future work is needed to determine key areas for consideration when attempting to conduct such an analysis. However, the case study demonstrates that where the concept of cure is clinically plausible and there is sufficient follow-up, MCM NMA can provide a method of comparative survival that acknowledges the benefit newer treatments may confer on a subset of patients resulting in long-term survival; this can be reflected in survival extrapolation and, later, in health economic modeling. In future, this method may provide an additional metric to compare treatments that is of value to patients.

## Supporting information

Chan et al. supplementary material Chan et al. supplementary material

## Data Availability

The data that support the findings of this study are available from the corresponding author, KC, upon reasonable request.

## Supplementary material

To view supplementary material for this article, please visit http://doi.org/10.1017/rsm.2025.10038.
