# Supplementary material for: Incorporating the possibility of cure into network meta-analyses: A case study from resected Stage III/IV melanoma
Source: Res Synth Methods. 2025 Oct 15;17(1):157–69. doi: 10.1017/rsm.2025.10038 (PMC12823198; doi:10.1017/rsm.2025.10038)
Supplement: Chan et al. supplementary material [file S1759287925100380sup001.docx]

# APPENDIX A: Transformations for survival functions

##### **Table 1: Parametric survival and hazard models, link functions, transformation to normal scale**

| **Distribution** | **Parameters** | **Link**  **function** | **Survival function** | **Hazard function** | **Transformation to normal scale** |
| --- | --- | --- | --- | --- | --- |
| **Exponential** | Rate ($\lambda$) | Log | $S(u)=exp(-\lambda u)$ | $h\left( u \right)=\lambda$  $\lambda>0$ | $\alpha_{1}=\log(\lambda)$ |
| **Weibull** | Shape ($\varphi$)  Scale ($\lambda$) | Log | $S(u)=exp(-\lambda u^{\varphi})$ | $h\left( u \right)=\lambda\varphi u^{(\varphi-1)}$  $\varphi>0 and \lambda>0$ | $\alpha_{1}=\log\left( \varphi\right)$  $\alpha_{2}=\log\left( \lambda\right)$ |
| **Log-normal** | Meanlog ($\lambda$)  sdlog ($\sigma$) | Identity | $S\left( u \right)=1-Ф\left( \frac{\log\left( u \right)-\lambda}{\sigma} \right)$  $\Phi$ is the CDF for standard normal distribution ($\lambda$*=*mean and $\sigma$*=*sd of variable's natural logarithm) | $h\left( u \right)=\frac{f(u)}{S(u)}$  $f(u)=\frac{1}{u\sigma\sqrt{2\lambda}}\exp\left( -\frac{1}{2}\left( \frac{\log\left( u \right)-\lambda}{\sigma} \right)^{2} \right)$ | $\alpha_{1}=\lambda$  $\alpha_{2}=\log\left( \sigma\right)$ |
| **Log-logistic** | Shape ($\theta$)  Scale ($\lambda$) | Log | $S(u)=\frac{1}{1+{(u/\lambda)}^{\theta}}$ | $h(u)=\frac{{(\theta}/{\lambda)}{(u/\lambda)}^{\theta-1}}{1+{(u/\lambda)}^{\theta}}$ | $\alpha_{1}=\log(\theta)$  $\alpha_{2}=\log\left( \lambda\right)$ |

***Abbreviations:*** *CDF, cumulative distribution function.*

# APPENDIX B: Summary of systematic literature review resected melanoma

## Adjuvant therapies for resected melanoma

The case study was based on a previously presented systematic review,^31^ current to May 2019, and involving an evidence synthesis of 52 reports representing 26 RCTs. Detailed reporting on the search strategy and conduct of the systematic review, along with characteristics of these 26 RCTs has been previously presented.^22,31^

For the current case study, the literature search was refreshed in June 2020, and the evidence base was restricted to RCTs according to eligibility criteria used in a previously presented conference poster:

- Population: resected stage III/IV melanoma;
- Interventions of interest: immunotherapies (nivolumab 3 mg/kg, ipilimumab 10 mg/kg, pembrolizumab 200 mg) or BRAF/MEK inhibitors (dabrafenib 150 mg + trametinib 2mg);
- Comparators: Placebo or best supportive care, any intervention of interest, any treatment that facilitates an indirect treatment comparison;
- Outcomes: RFS; and
- Language: restricted to publications in English.

The PRISMA flow diagram for the latest update to the adjuvant melanoma SLR informing the case study is presented in **Figure 1**, which includes the reduction to 4 RCTs according to the eligibility criteria for the case study. Summary of the included studies is provided in **Table 2**.

Figure 1: PRISMA flow diagram for latest update to the systematic literature review of adjuvant therapies for resected melanoma, informing the case study


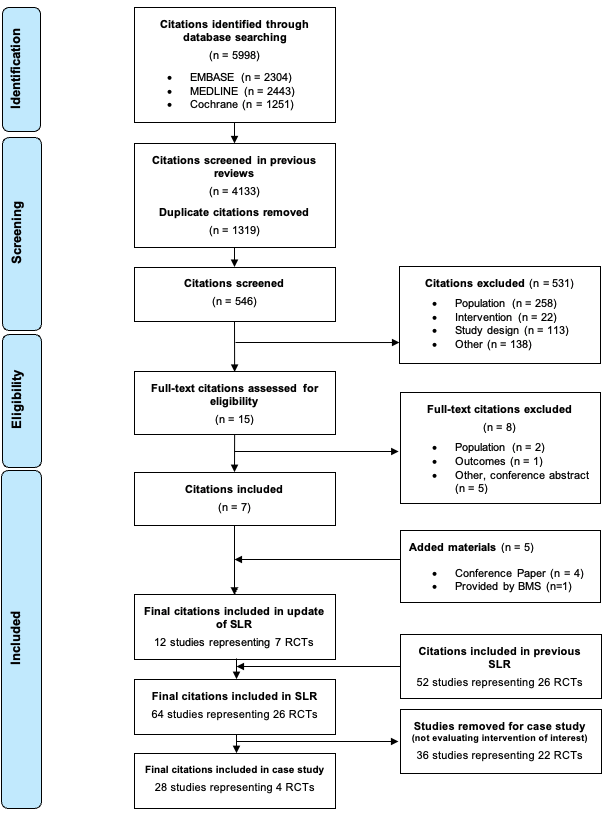


##### **Table 2: Summary of trials identified in the systematic literature review of adjuvant therapies for resected melanoma**

| **Trial ID** | **Primary Publication** | **NCT Code** | **Blinding** | **Phase** | **Treatment 1 (sample size)** | **Treatment 2 (sample size)** | **RFS for Treatment 1 vs. Treatment 2, HR (95% CI)** |
| --- | --- | --- | --- | --- | --- | --- | --- |
| EORTC 18071 | Eggermont et al 2019^40^ | NCT00636168 | Double-blind | III | Ipilimumab (n=476) | Placebo (n=475) | 0.75 (0.63, 0.88) |
| Keynote 054 | Eggermont et al 2019^41^ | NCT02362594 | Double-blind | III | Pembrolizumab (n=502) | Placebo (n=509) | 0.56 (0.43, 0.74)* |
| COMBI-AD | Hauschild et al 2019^42^ | NCT01682083 | Double-blind | III | Dabrafenib +Trametinib (n=432) | Placebo (n=438) | 0.51 (0.42, 0.61) |
| CheckMate 238 | Weber et al 2017^43^ | NCT02388906 | Double-blind | III | Nivolumab (n=452) | Ipilimumab (n=453) | 0.68 (0.56, 0.82) |

***Note:*** *Data is presented only from the most recent data source for each trial; * – 98.4% confidence interval.* ***Abbreviations:*** *CI, confidence interval; HR, hazard ratio; RFS, recurrence-free survival.*

##### **Table 3: Key study and patient characteristics of trials identified in the systematic literature review of adjuvant therapies for resected melanoma**

| **Trial name** | **Treatment arm** | **Mean age (range)** | **% Male** | **Countries** |
| --- | --- | --- | --- | --- |
| EORTC 18071 | Ipilimumab | 51 (20–84) | 62 | France, Denmark, Netherlands, Italy, United States of America, Australia, Russian Federation, Czech Republic, Finland, Spain, Sweden, United Kingdom, Poland, Norway, Canada, Belgium, Germany, Switzerland, Austria |
|  | Placebo | 52 (18–78) | 62 |  |
| Keynote 054 | Pembrolizumab | 54 (19–88) | 63 | France, Netherlands, Australia, Italy, United States of America, United Kingdom, Canada, New Zealand, Belgium, Germany, Russian Federation, Poland, Israel, Norway, Finland, Denmark, Switzerland, Spain, Portugal, Japan, Serbia, Sweden |
|  | Placebo | 54 (19–83) | 60 |  |
| COMBI-AD | Dabrafenib plus trametinib | 50 (18-89) | 56 | United States of America, Argentina, Australia, Austria, Belgium, Brazil, Canada, Czechia, Denmark, France, Germany, Greece, Israel, Italy, Japan, Netherlands, New Zealand, Norway, Poland, Russian Federation, Spain, Sweden, Switzerland, Taiwan, United Kingdom |
|  | Placebo | 51 (20-85) | 55 |  |
| CheckMate 238 | Nivolumab | 56 (19–83) | 57 | United States of America, Argentina, Australia, Austria, Belgium, Canada, Czechia, Finland, France, Greece, Hungary, Ireland, Italy, Japan, Korea (Republic of), Netherlands, Norway, Poland, Romania, South Africa, Spain, Sweden, Switzerland, Taiwan, United Kingdom |
|  | Ipilimumab | 54 (18–86) | 59 |  |

# APPENDIX C: JAGS model code

Model was run using 20,000 initial samples which were discarded (i.e. burn-in) and thinning of the sampling was set to 2 (i.e. every 2^nd^ sample is discarded). Convergence of the posterior distribution were assessed using density, trace and Gelman–Rubin diagnostic plots.

model { #model 2b, 3 parameters

# i = data point (one for each arm of each study),

# arm = study arm

# s = study

# m = parameter

#Likelihood for arm level data

#=======================

for(i in 1:N1){

tmp1[i] <- studyid[i] # study id not used in the model

y[i,1:3] ~ dmnorm(mean.y[study[i],arm[i],1:3],omega[i,,]) # multivariate likelihood

omega[i,1:3,1:3] <- inverse(cov.mat[i,,]) # within-study precision matrix

#define elements of within-study covariance matrix

cov.mat[i,1,1] <- cov11[i]

cov.mat[i,2,2] <- cov22[i]

cov.mat[i,3,3] <- cov33[i]

cov.mat[i,1,2] <- cov12[i]

cov.mat[i,1,3] <- cov13[i]

cov.mat[i,2,3] <- cov23[i]

cov.mat[i,2,1] <- cov.mat[i,1,2]

cov.mat[i,3,1] <- cov.mat[i,1,3]

cov.mat[i,3,2] <- cov.mat[i,2,3]

}

for(j in 1:ns){

for(k in 1:nastudy[j]) {

for(m in 1:no){

mean.y[j,k,m] <- mu[j,m] + delta[j,k,m] # define study-specific treatment effects

}

}

}

#Fixed effects between-study model

#=================================

for(j in 1:ns) {

for(m in 1:no) {

delta[j,1,m] <-0 # delta in control arm to zero for all outcomes

}

for(k in 2:nastudy[j]){

for(m in 1:no) {

delta[j,k,m] <- md[j,k,m] #fixed effects model

}

}

}

#Consistency relations between basic parameters

#=====================================

for(i in 1:N2) {

tmp2[i] <- studyid1[i] # temp variable to identify study id, not used

for(k in 2:na[i]) {

md[s[i],k,out[i]] <- (d[out[i],t[i,k]] - d[out[i],t[i,1]])*equals(o[i],out[i])

}

}

#Constraints

# Effect in reftx is set to zero

d[1,1] <- 0

d[2,1] <- 0

d[3,1] <- 0

for(m in 1:no) {

for(j in 1:ns){

mu[j, m] ~ dnorm(0,0.001)

}

for(k in 2: nt.total[m]){

or[m,k] <- exp(d[m,k])

d[m,k] ~ dnorm(0,0.001)

}

}

#spherical parameterization (Wei and Higgins 2013)

for (i in 1: ns){

mu1[i,1] <- mean.y[i,1,1]*equals(t[(i*no),1],1)

mu1[i,2] <- mean.y[i,1,2]*equals(t[(i*no),1],1)

mu1[i,3] <- mean.y[i,1,3]*equals(t[(i*no),1],1)

st1[i] <- equals(t[(i*no),1],1)

}

mu_mean[1]<- sum(mu1[,1])/sum(st1[])

mu_mean[2]<- sum(mu1[,2])/sum(st1[])

mu_mean[3]<- sum(mu1[,3])/sum(st1[])

for (i in 1: nt.total[1]){

alpha[1,i] <- mu_mean[1]+d[1,i]

}

for (i in 1: nt.total[2]){

alpha[2,i] <- mu_mean[2]+d[2,i]

}

for (i in 1: nt.total[3]){

alpha[3,i] <- mu_mean[3]+d[3,i]

}

}

# APPENDIX D: Modeled background mortality

##### **Table 4: Statistical goodness of fit to background mortality data in RCTs involving adjuvant therapies for resected melanoma**

| **Trial** | **Akaike information criterion** | | | | |
| --- | --- | --- | --- | --- | --- |
|  | **Weibull** | **Gompertz** | **Log-normal** | **Log-logistic** | **Exponential** |
| CheckMate 238 | 3687.6 | 3595.1 | 4074.2 | 3926.1 | 4017.7 |
| COMBI-AD | 4034.0 | 3895.9 | NA | 4315.7 | 4574.1 |
| EORTC 18071 | 3960.3 | 3847.3 | 4392 | 4221.3 | 4397.7 |
| KEYNOTE-054 | 4037.5 | 3909.6 | NA | 4319.4 | 4459.0 |

##### **Figure 2: Visual depiction of goodness of fit to background mortality data in RCTs involving adjuvant therapies for resected melanoma**


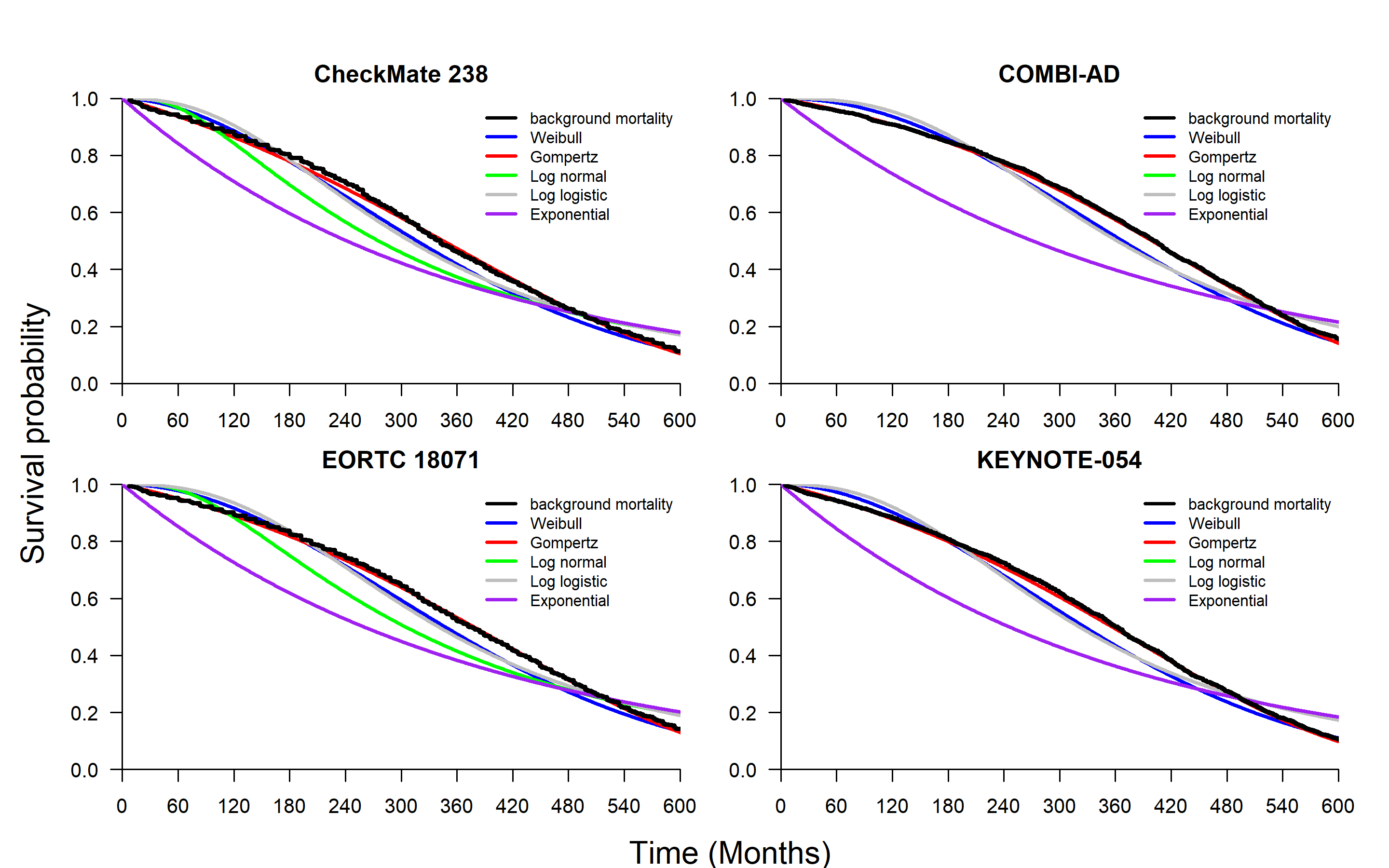


# APPENDIX E: Survival data informing analyses and fitted mixture cure models

Across the MCMs fit to the RFS data, the parametric shape with the lowest (best) aggregate AIC was the MCM involving a log-normal parametric form for modeling uncured survival (**Table 5**).

##### **Table 5: Statistical goodness of fit to all trial arms in the network of evidence of adjuvant therapies for resected melanoma**

| **Trial** | **Treatment** | **Akaike information criterion** | | | |
| --- | --- | --- | --- | --- | --- |
|  |  | **Weibull** | **Log-normal** | **Log-logistic** | **Exponential** |
| CheckMate-238 | IPI | 2140.7 | 2127.8 | 2123.5 | 2140.2 |
|  | NIVO | 1841.1 | 1823.3 | 1824.6 | 1841.4 |
| COMBI-AD | DAB+TRAM | 1864.6 | 1870.4 | 1863.5 | 1902.3 |
|  | Placebo | 2200.9 | 2157.1 | 2169.3 | 2202.4 |
| EORTC 18071 | IPI | 2689.1 | 2673.7 | 2677 | 2687.8 |
|  | Placebo | 2927.5 | 2918.6 | 2913.8 | 2926.1 |
| KEYNOTE-054 | PEM | 1550.5 | 1540.4 | 1543.8 | 1548.7 |
|  | Placebo | 2130.6 | 2114.7 | 2119.3 | 2139.9 |
| **Aggregate AIC** | | **17345** | **17226** | **17234.8** | **17388.8** |

The log-normal model was best-fitting in five of the eight trial arms; in the three trial arms where the log-normal parametric form (best according to aggregate AIC) was not the best-fitting parametric form, the AIC was not substantially worse than the better-fitting model (at most 6.9 units in the DAB+TRAM arm of the COMBI-AD trial), and the cure fraction estimates were stable (**Figure 3**). Additionally, the visual inspections of the modeled survival overlayed with the observed KM curves (**Figure 4**) and the residuals (observed KM survival – modeled survival) (**Figure 5**) showed that the log-normal model was reasonably fit to the observed data, with clinically plausible projections.

Across all trial arms, and parametric forms for uncured survival, the cure fraction estimates were relatively stable, apart from the exponential fit to the DAB+TRAM arm of the COMBI-AD trial, in which the cure fraction up to 20% lower than with other parametric forms (**Figure 3)**. However, the AIC for this exponential fit was nearly 40 units higher (worse) than the best fitting model, and over 30 units higher (worse) than with the log-normal model; furthermore, visual inspections (**Figure 4**) showed that the exponential model was poorly fit to the observed trial data.

As a result, the log-normal model was selected as the parametric form for modeling uncured survival within the MCMs of the main analysis.

##### **Figure 3: Assessment of cure fraction sensitivity to mixture cure model parametric shape and associated statistical goodness of fit by trial arm; adjuvant therapies for resected melanoma**


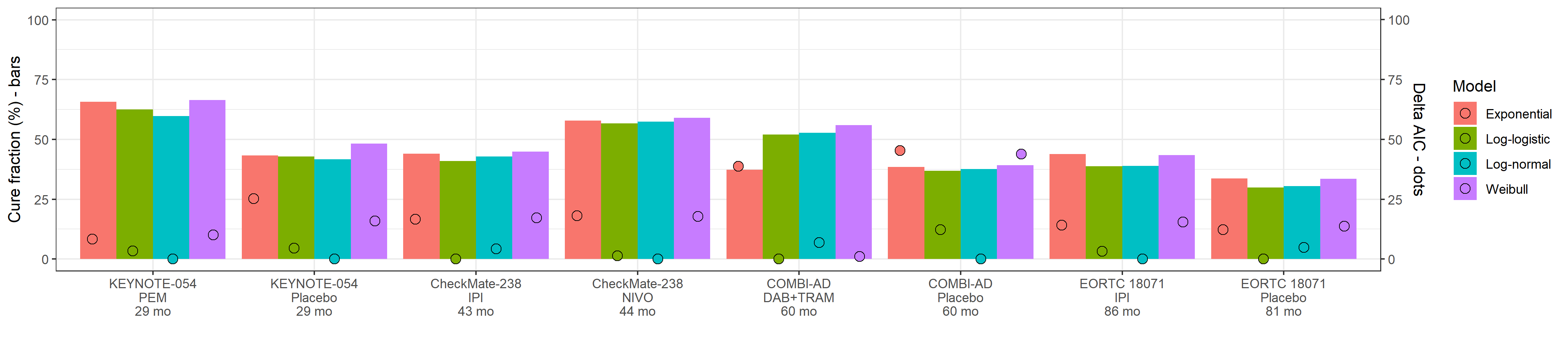
***Note:*** *Delta AIC was calculated as the AIC of each model minus the AIC of the best-fitting model; for the best fitting model, the delta AIC is therefore equal to zero.*

##### **Figure 4: Assessment of cure fraction sensitivity to mixture cure model parametric shape and associated statistical goodness of fit by trial arm; adjuvant therapies for resected melanoma**


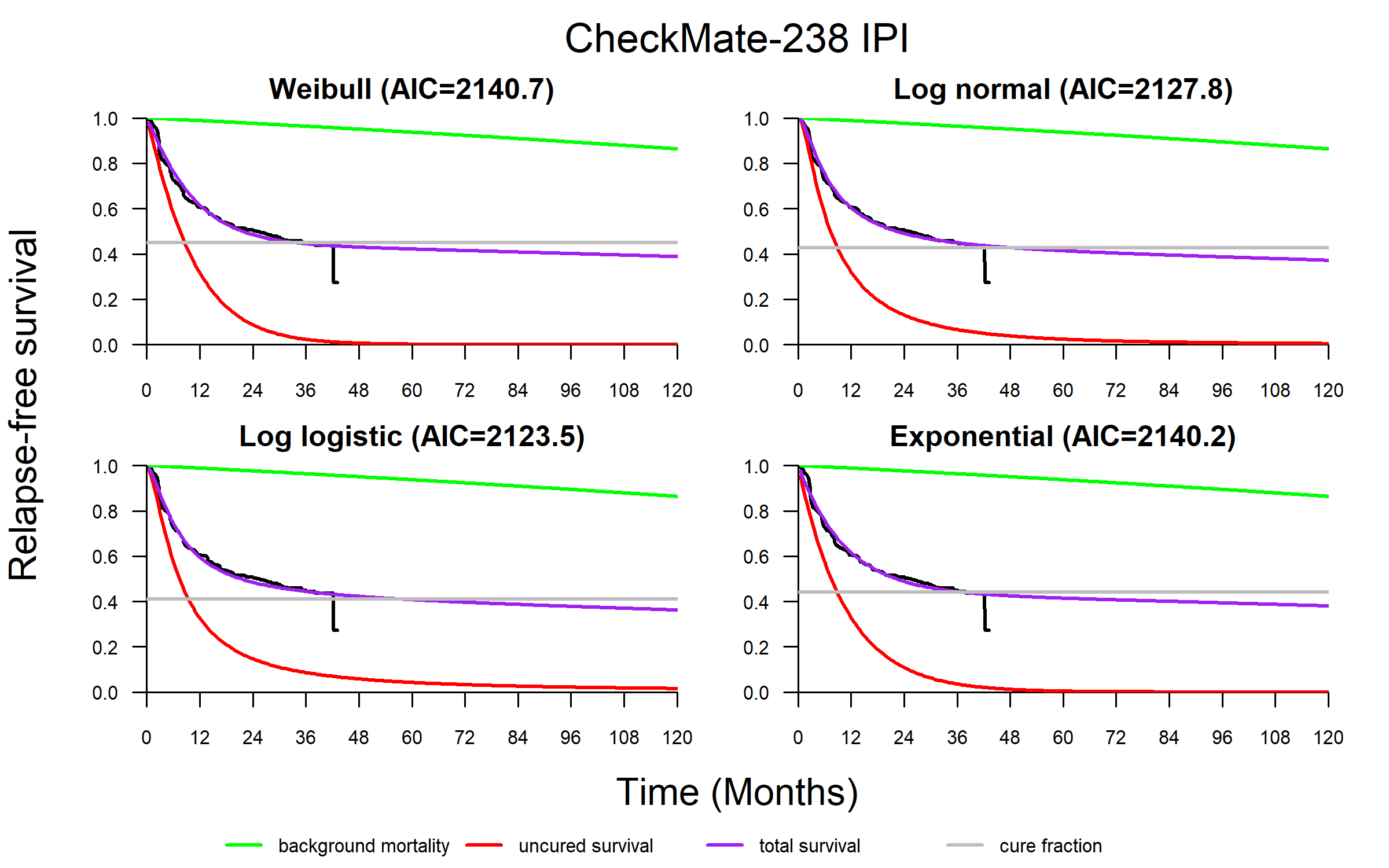

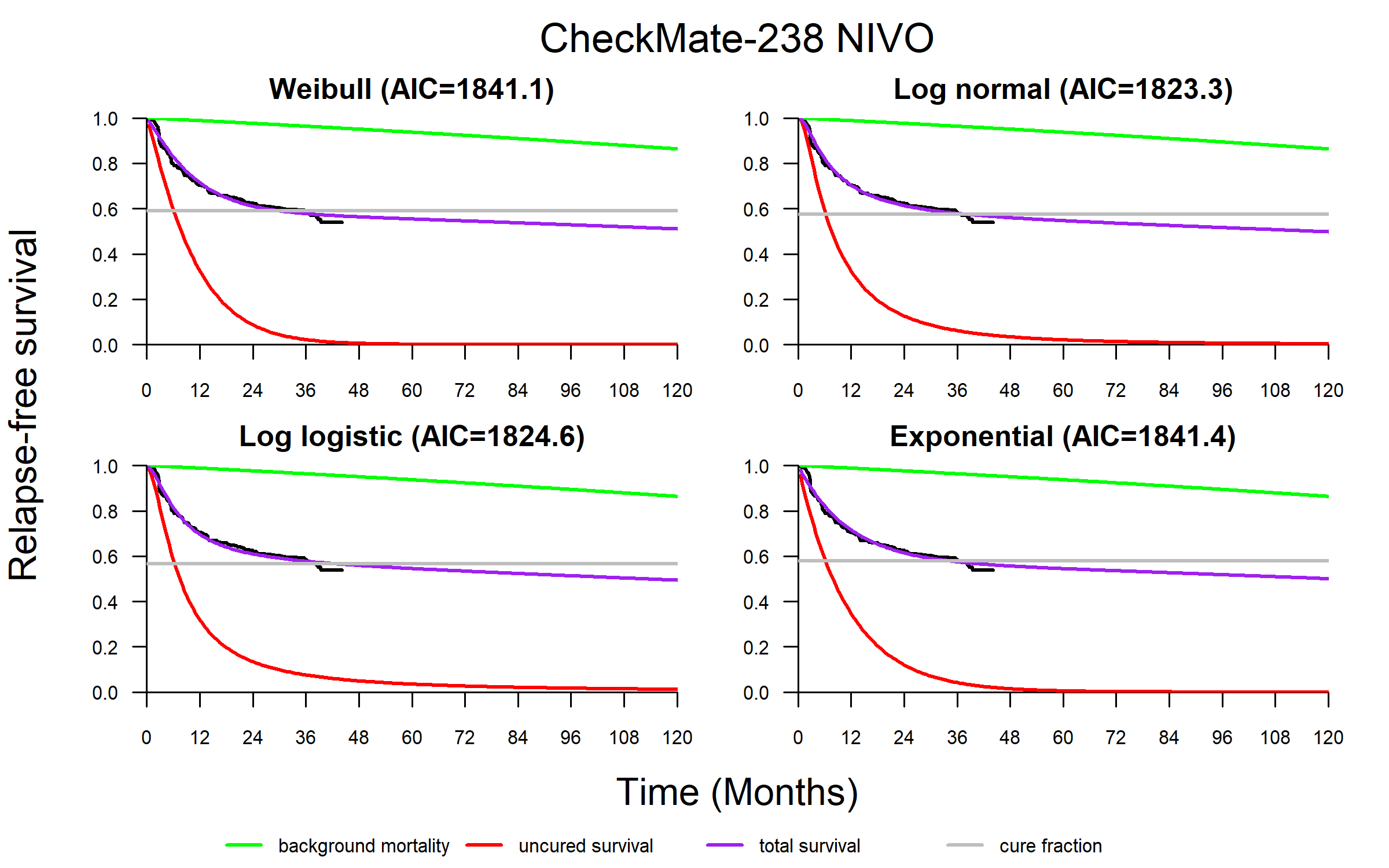


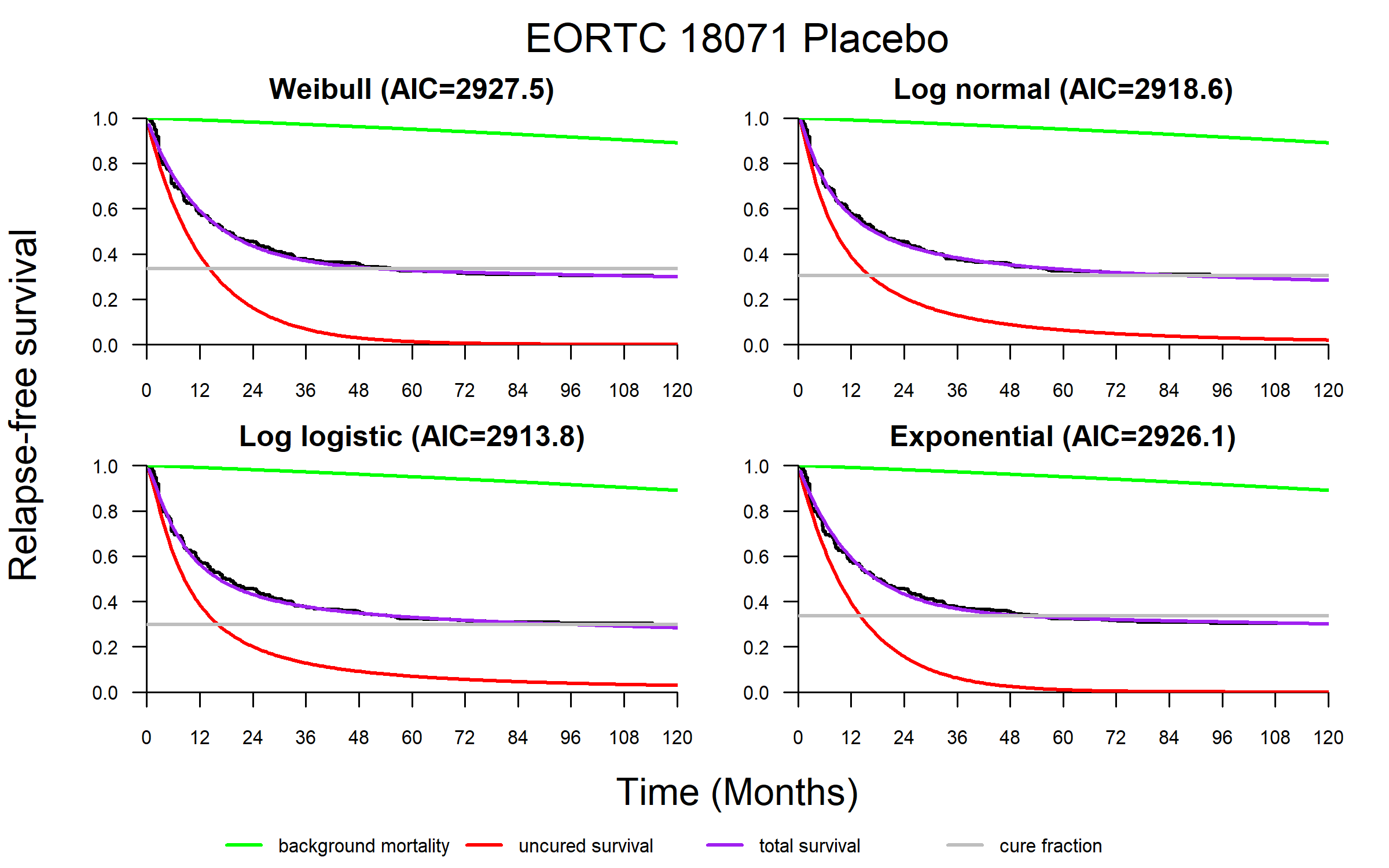


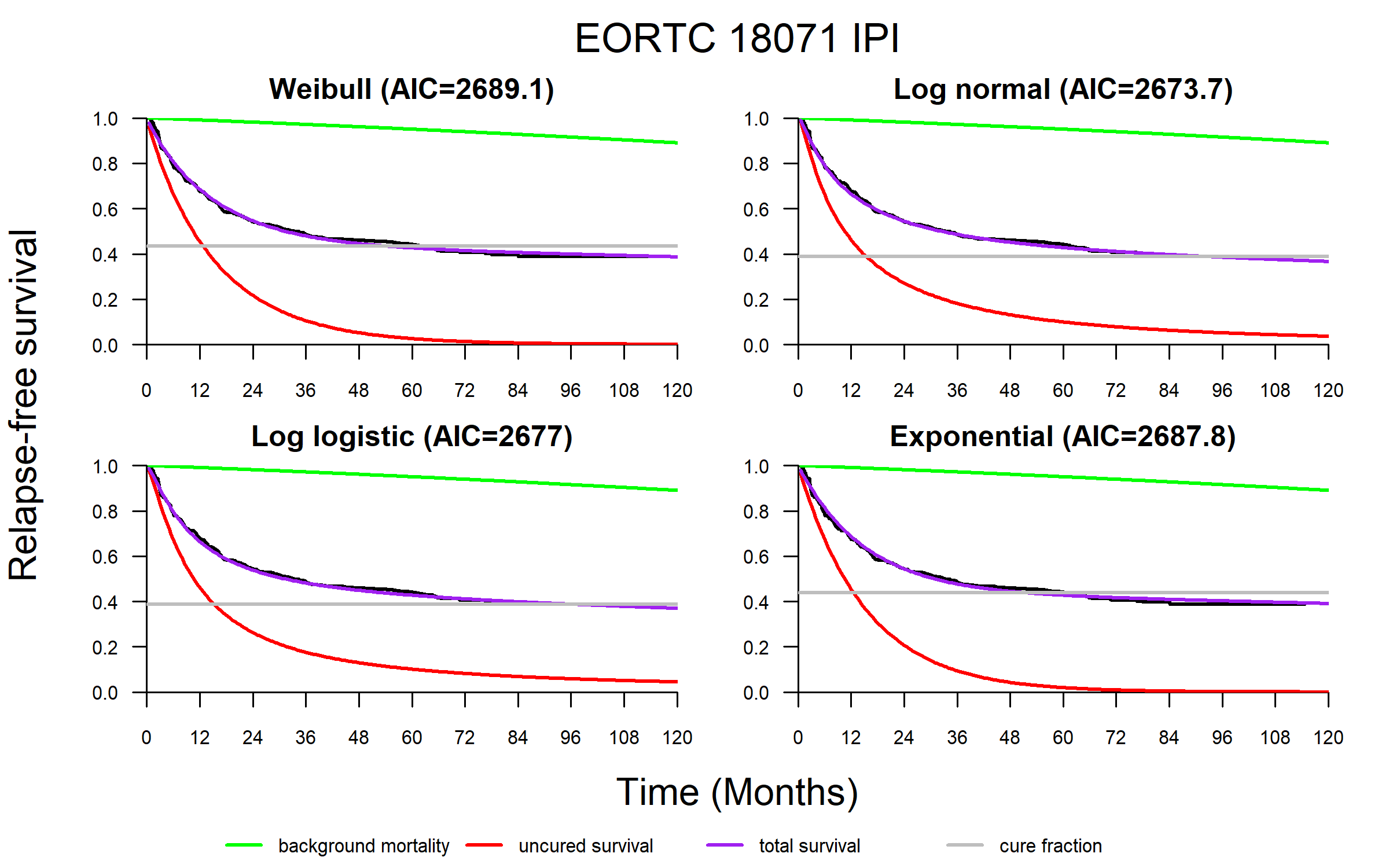


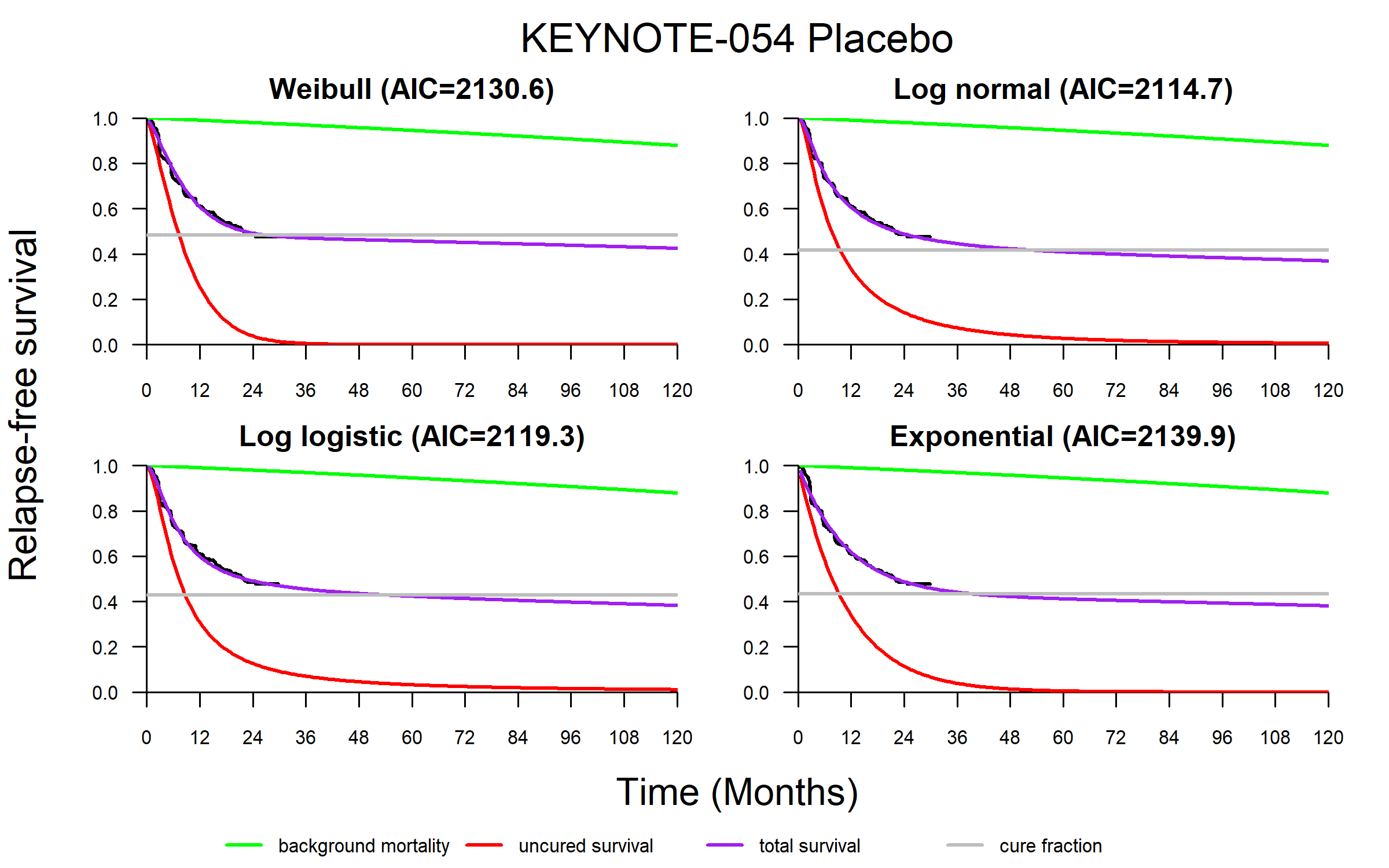

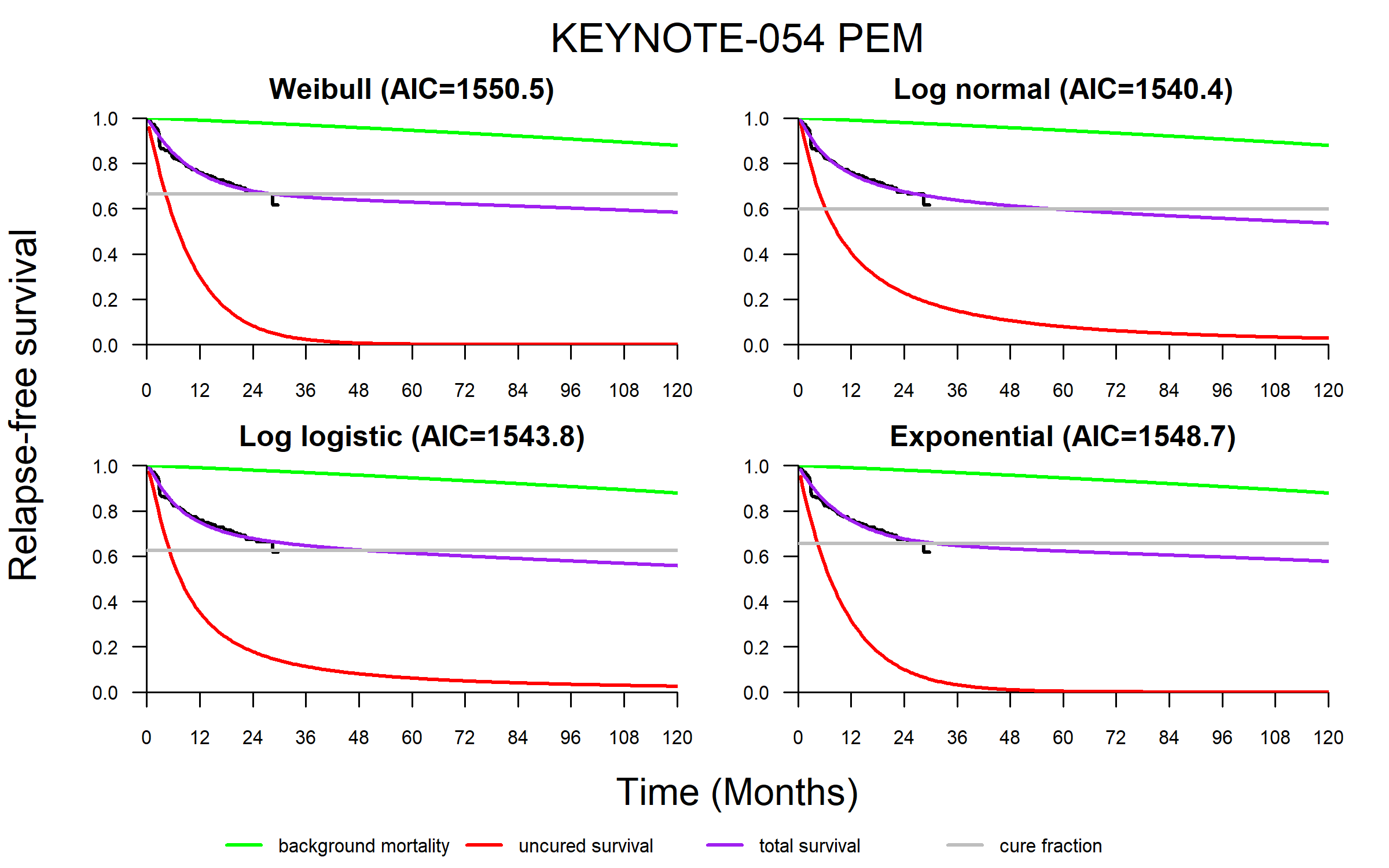


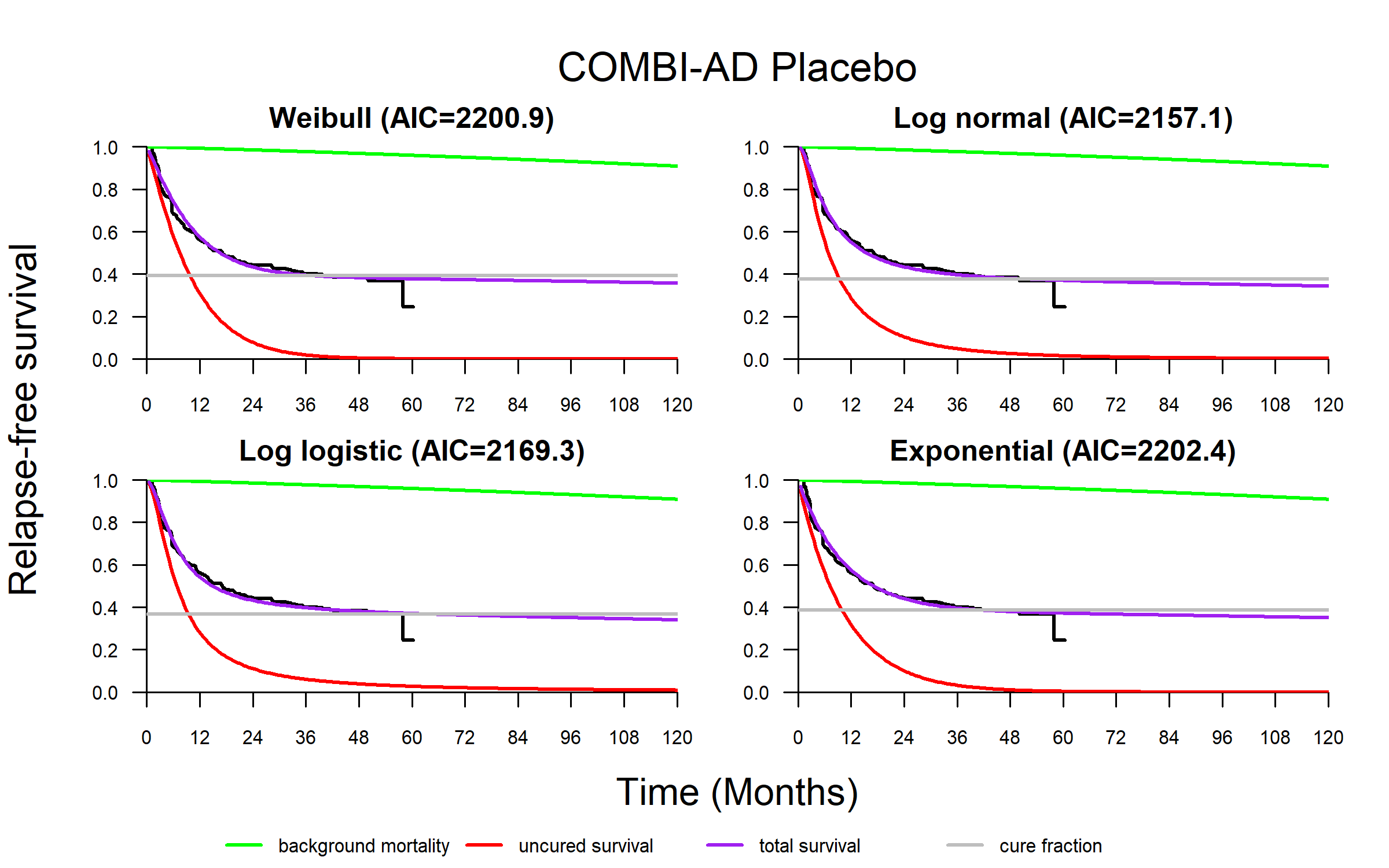

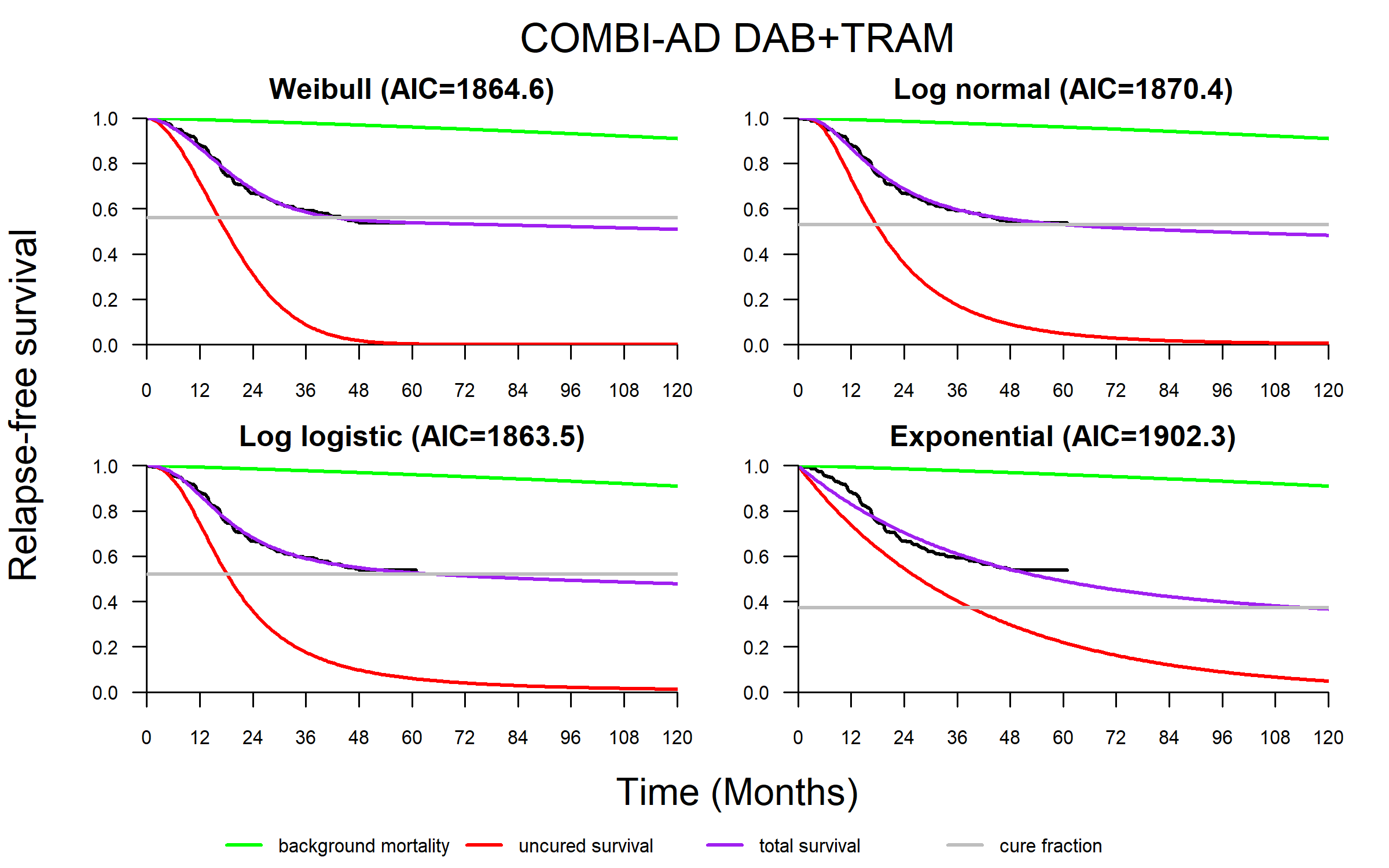


##### **Figure 5: Assessment of mixture cure model residuals and associated statistical goodness of fit by trial arm; adjuvant therapies for resected melanoma**


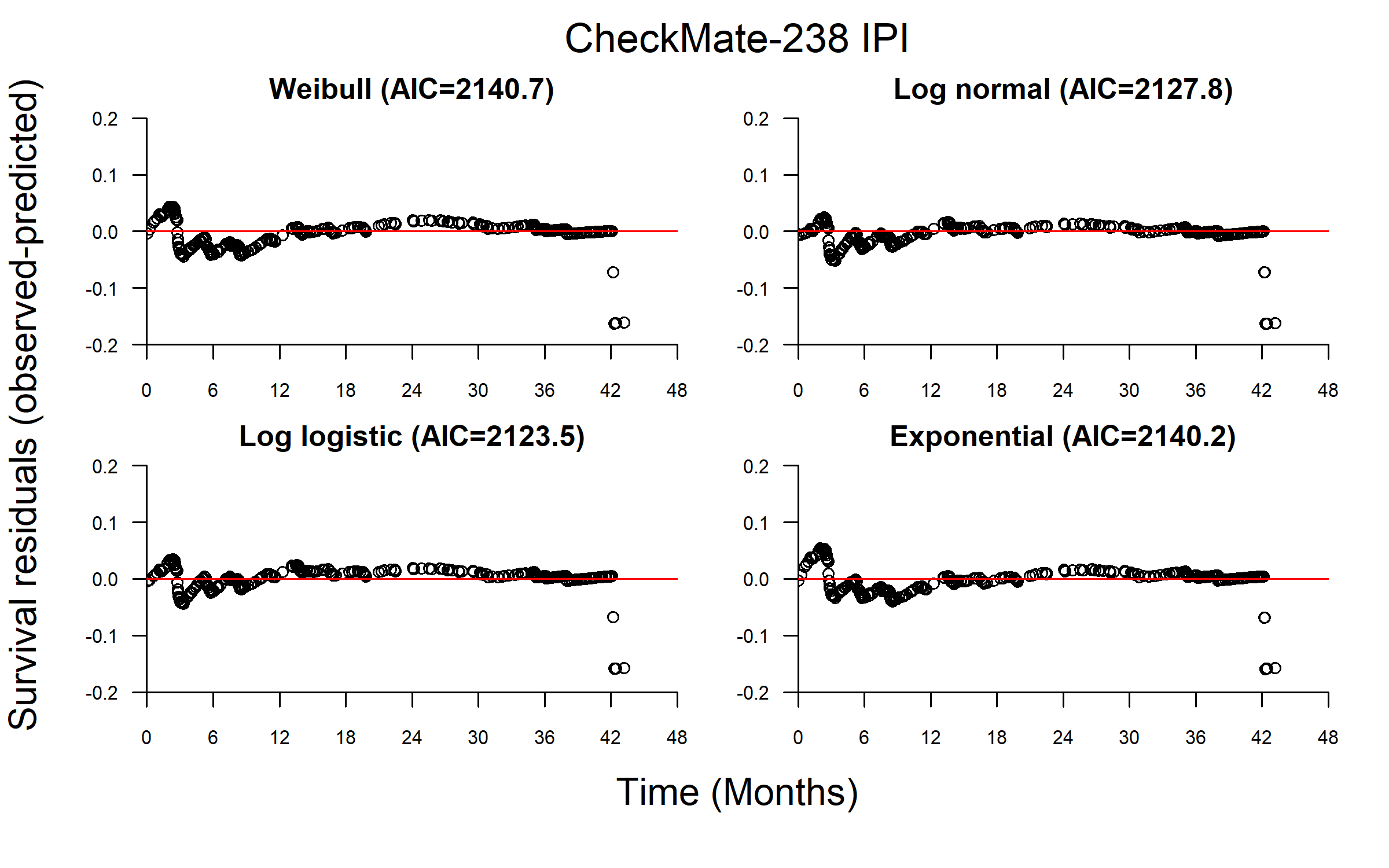


**
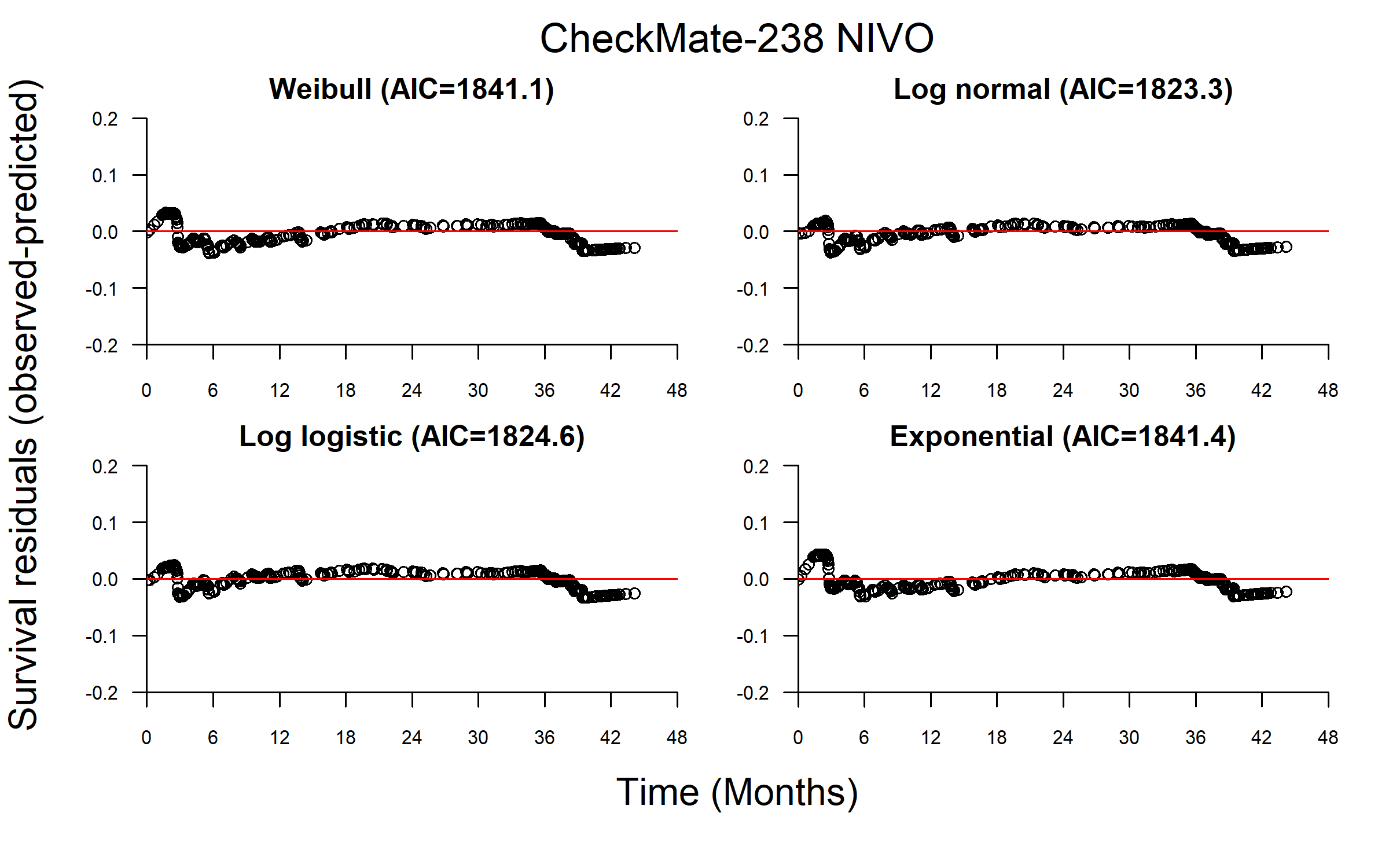
**

**
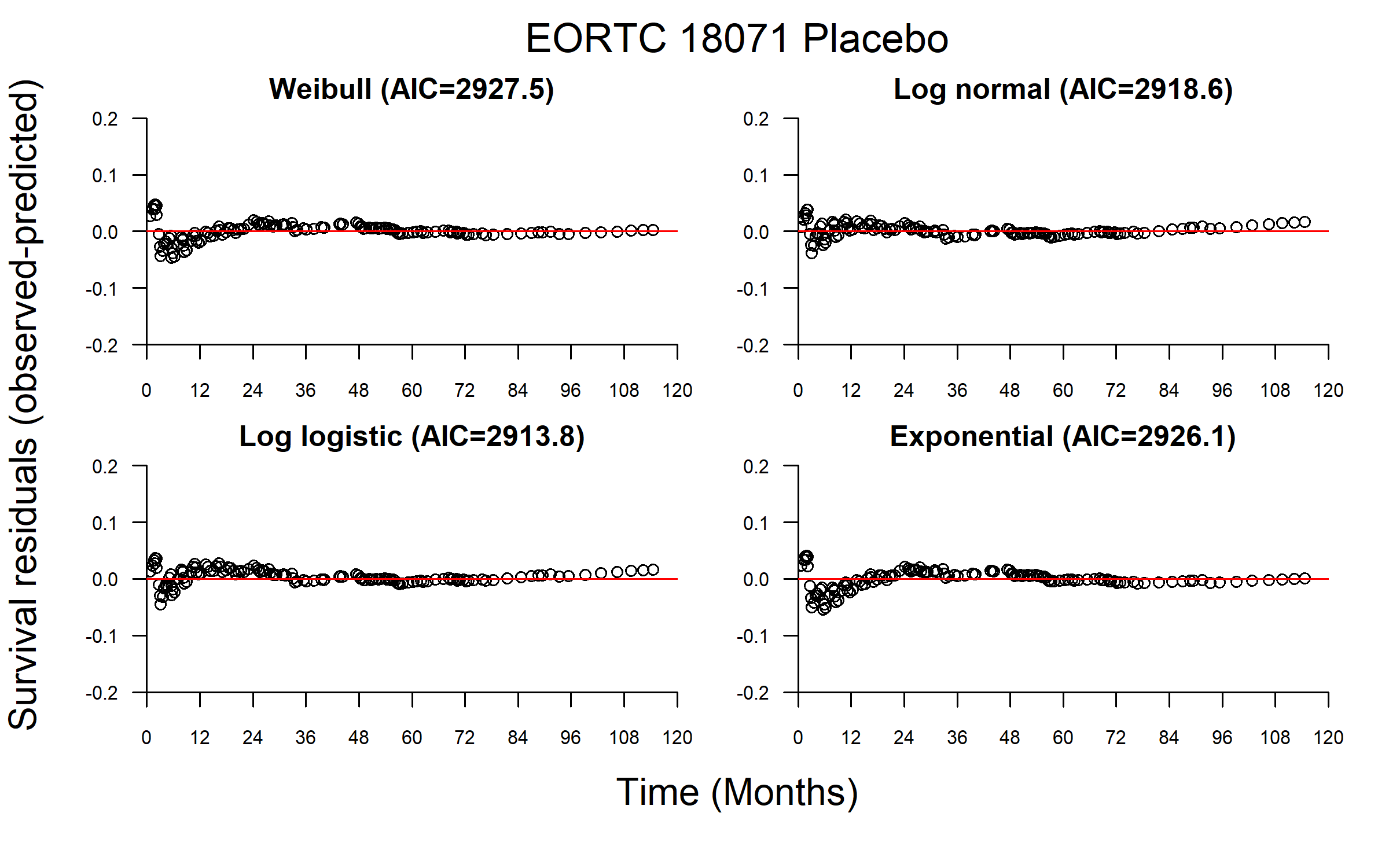
**

**
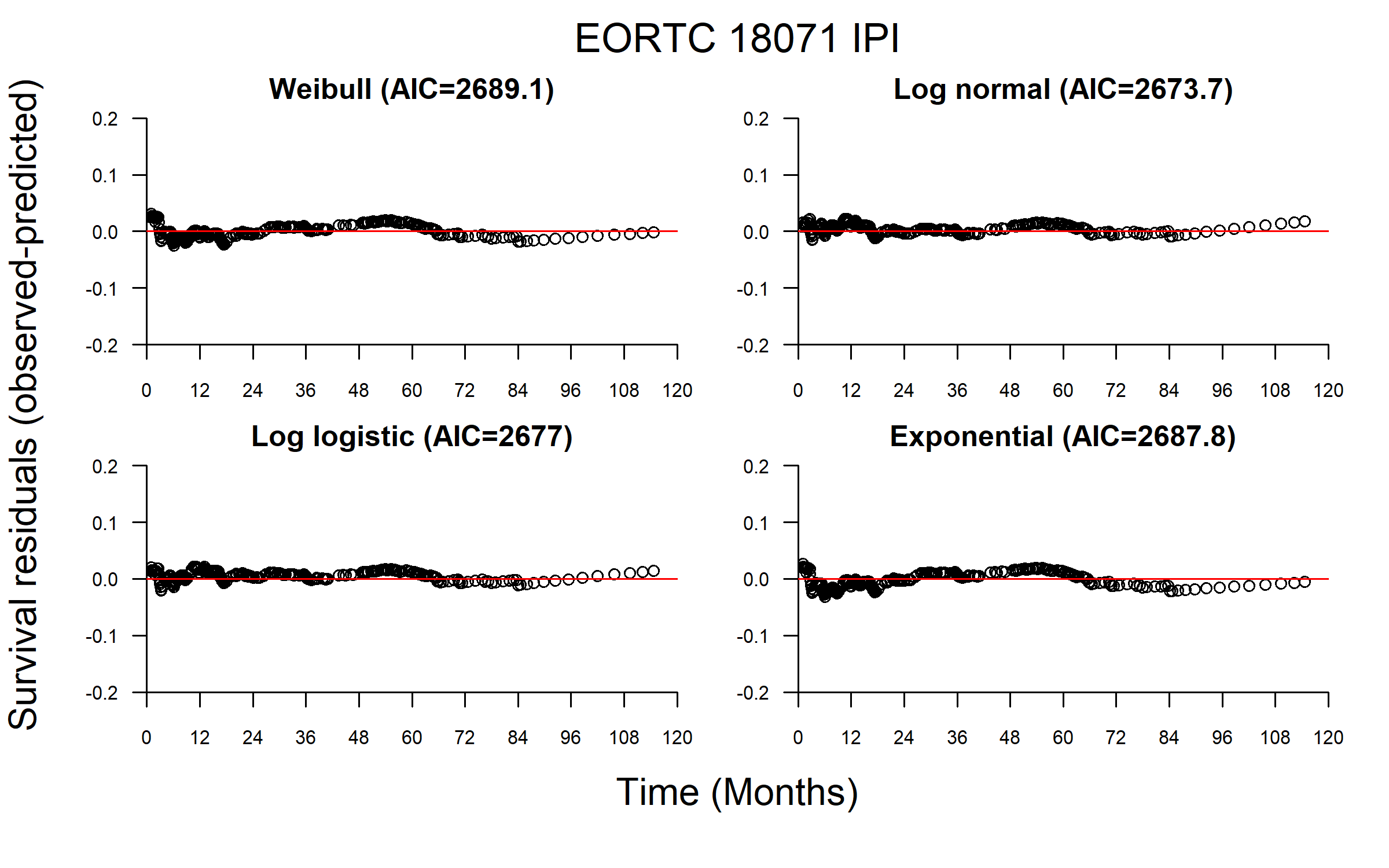
**

**
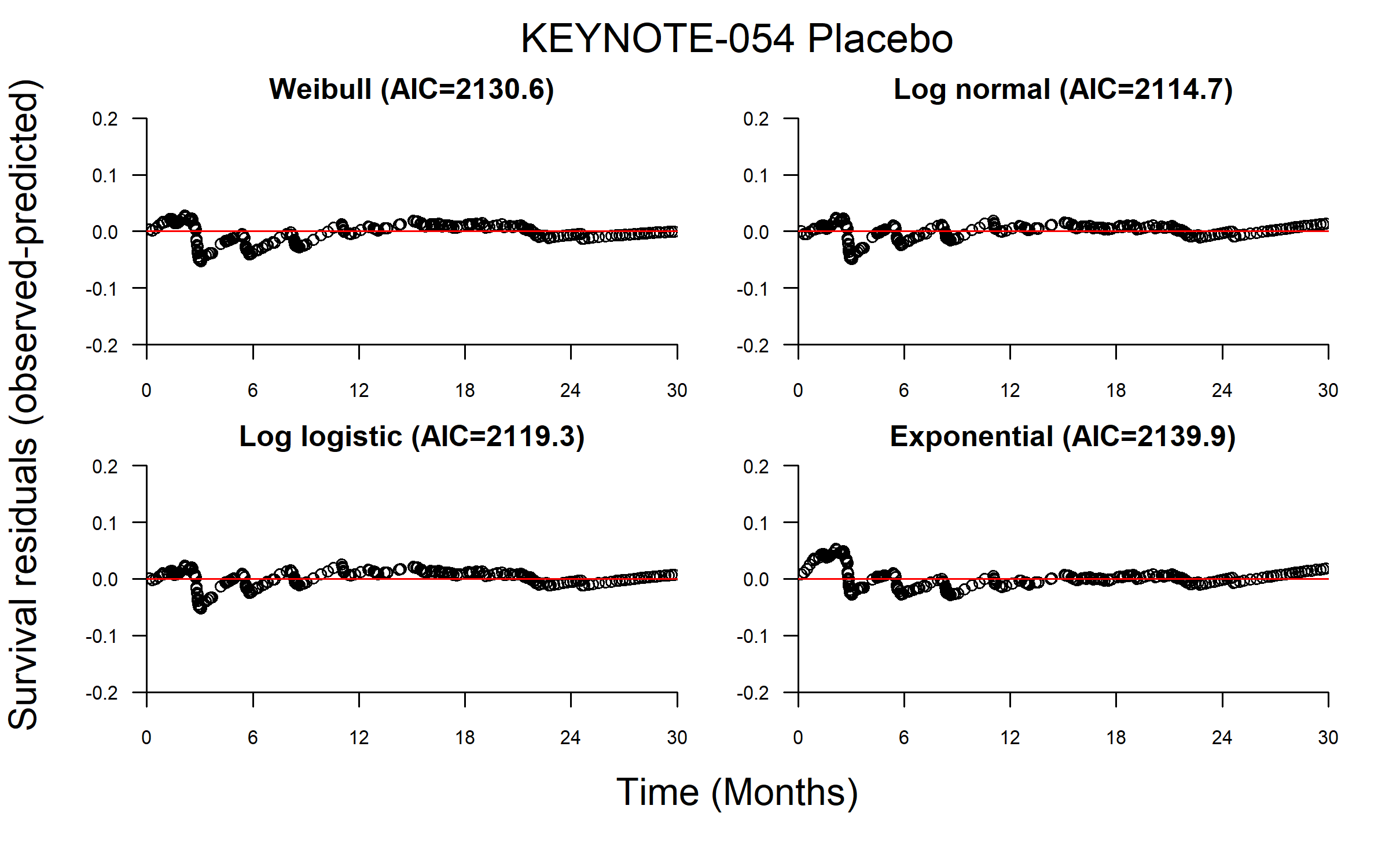
**

**
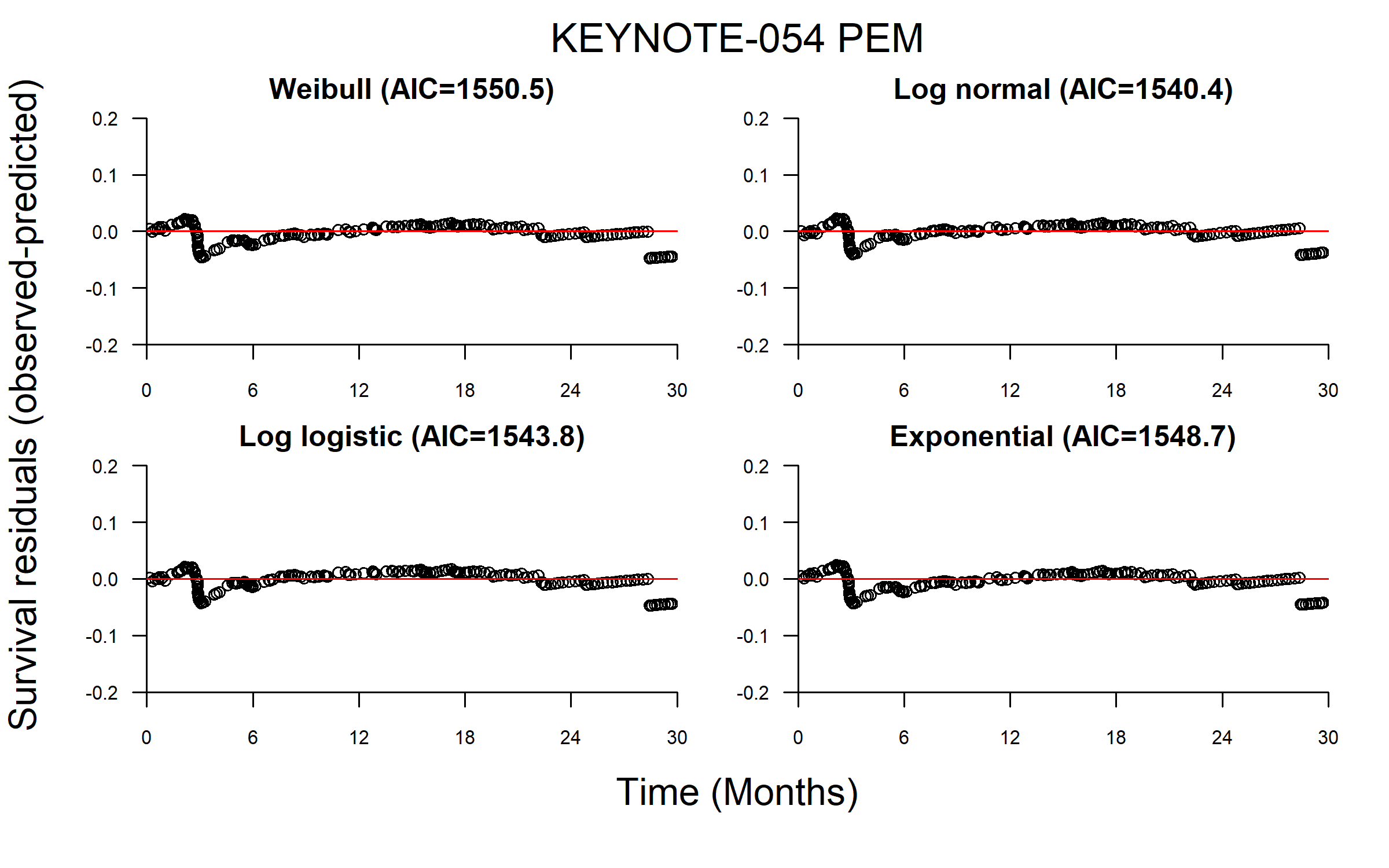
**

**
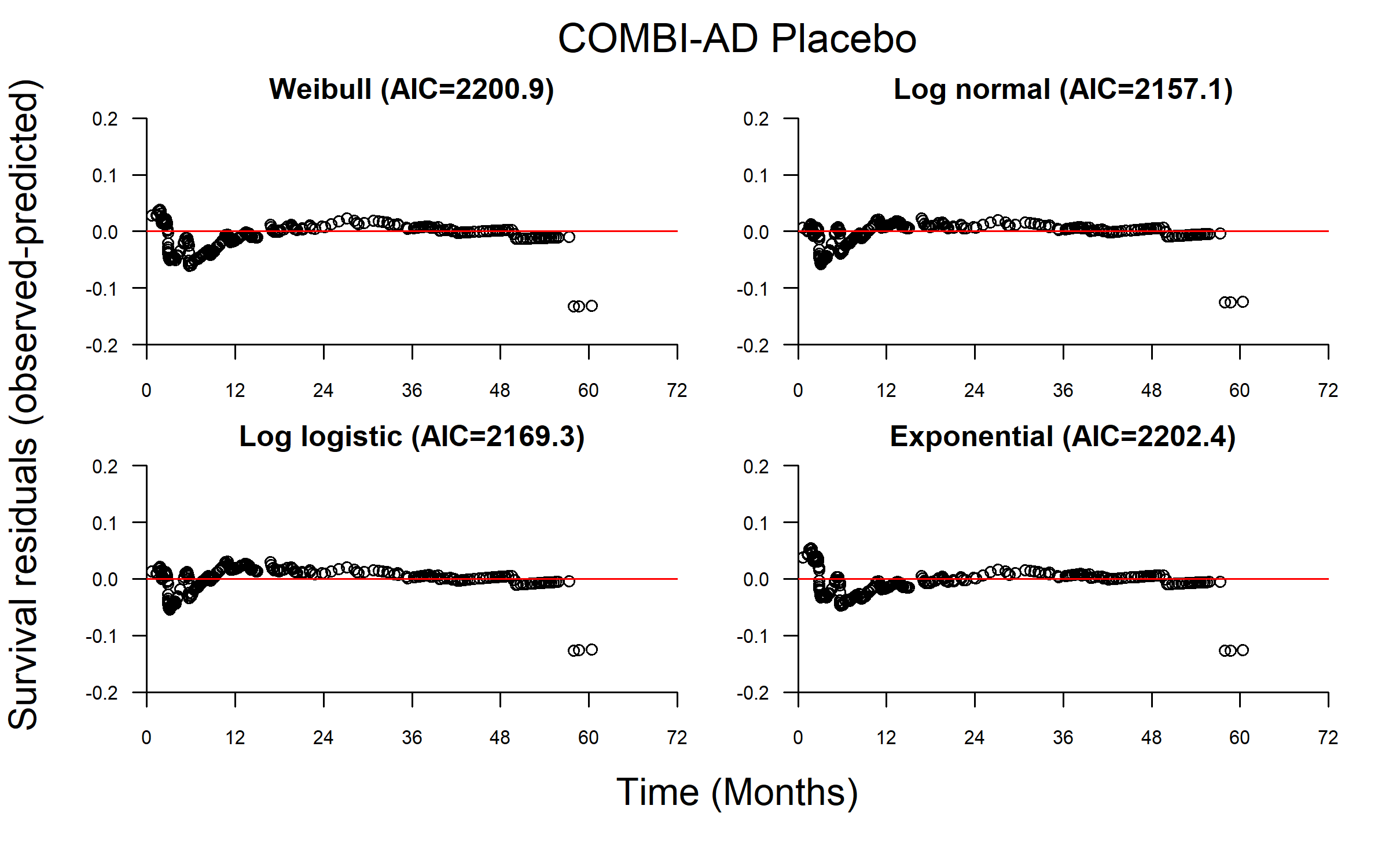
**

**
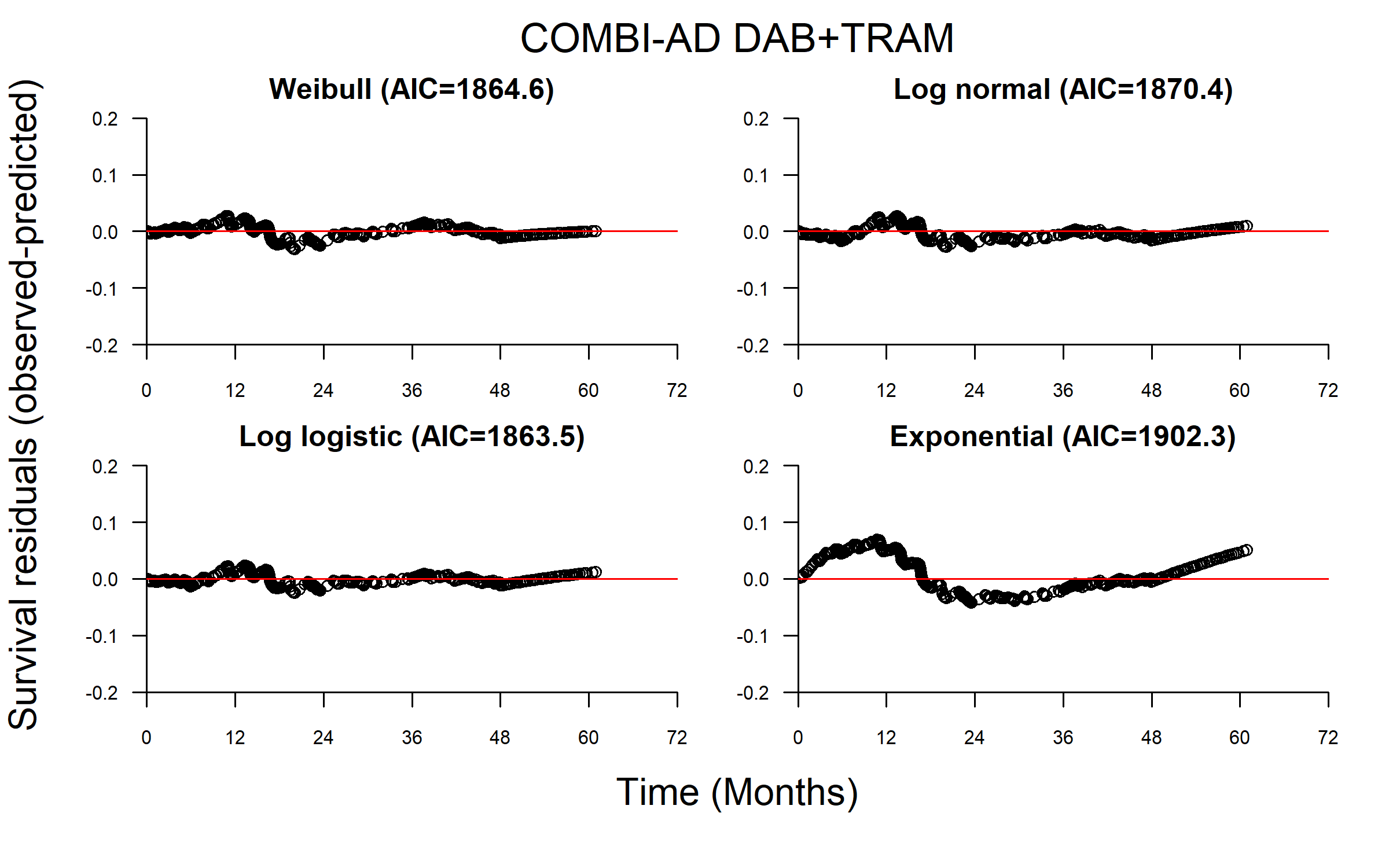
**

# APPENDIX F: Bayesian MCM NMA model inputs and relative effect outputs

##### **Table 6: MCM NMA model inputs; adjuvant therapies for resected melanoma**

| **Trial** | **Treatment** | **alpha1** | **alpha2** | **alpha3** | **cov11** | **cov12** | **cov13** | **cov22** | **cov23** | **cov33** |
| --- | --- | --- | --- | --- | --- | --- | --- | --- | --- | --- |
| CheckMate 238 | IPI | -0.290 | 1.992 | 0.054 | 0.017 | -0.006 | -0.005 | 0.009 | 0.004 | 0.006 |
|  | NIVO | 0.300 | 2.022 | 0.012 | 0.015 | -0.006 | -0.005 | 0.012 | 0.005 | 0.007 |
| EORTC 18071 | IPI | -0.450 | 2.355 | 0.303 | 0.019 | -0.009 | -0.005 | 0.015 | 0.005 | 0.004 |
|  | Placebo | -0.825 | 2.124 | 0.254 | 0.017 | -0.004 | -0.004 | 0.008 | 0.002 | 0.004 |
| KEYNOTE-054 | Placebo | -0.336 | 2.024 | 0.071 | 0.030 | -0.016 | -0.009 | 0.016 | 0.007 | 0.006 |
|  | PEM | 0.397 | 2.160 | 0.313 | 0.055 | -0.056 | -0.022 | 0.078 | 0.027 | 0.014 |
| COMBI-AD | DAB+TRAM | 0.113 | 2.922 | -0.347 | 0.020 | -0.006 | -0.007 | 0.007 | 0.004 | 0.009 |
|  | Placebo | -0.506 | 1.926 | -0.005 | 0.014 | -0.002 | -0.002 | 0.006 | 0.002 | 0.003 |

**Abbreviations:** DAB+TRAM – combination dabrafenib and trametinib; IPI – ipilimumab; NIVO – nivolumab; PEM –pembrolizumab

##### **Table 7: Relative treatment effect estimate outputs for cure fraction odds ratios (with corresponding 95% credible interval); adjuvant therapies for resected melanoma**

| **Placebo** | **0.69  (0.48, 1.00)** | **0.54  (0.37, 0.77)** | **0.48  (0.27, 0.85)** | **0.38  (0.23, 0.64)** |
| --- | --- | --- | --- | --- |
| **1.45  (1.00, 2.09)** | **IPI** | 0.78  (0.47, 1.31) | 0.69  (0.35, 1.38) | **0.56  (0.39, 0.79)** |
| **1.86  (1.30, 2.68)** | 1.28  (0.77, 2.15) | **DAB + TRAM** | 0.89  (0.46, 1.75) | 0.71  (0.38, 1.33) |
| **2.10  (1.18, 3.68)** | 1.44  (0.73, 2.83) | 1.12  (0.57, 2.20) | **PEM** | 0.80  (0.37, 1.72) |
| **2.61  (1.57, 4.36)** | **1.80  (1.27, 2.56)** | 1.40  (0.75, 2.63) | 1.25  (0.58, 2.70) | **NIVO** |

***Note:*** *Each cell represents the comparison (odds ratio and 95% CrI) of the row treatment versus the column treatment where an odds ratio >1 indicates higher odds of cure for the row treatment versus the column treatment; All bolded values are statistically meaningful at the 0.05 significance level.* ***Abbreviations:*** *DAB+TRAM, combination dabrafenib and trametinib; IPI, ipilimumab; NIVO, nivolumab; PEM, pembrolizumab.*

##### **Figure 6: Relative treatment effect estimate outputs for recurrence-free survival among the uncured, as hazard ratios relative to observation/placebo (with corresponding 95% credible interval); adjuvant therapies for resected melanoma**


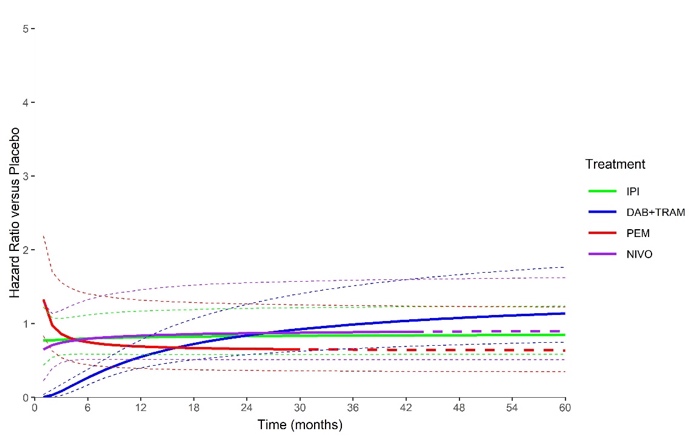


***Note:*** *Thick dashed lines indicate estimates are based on extrapolation; thin dashed lines are the 95% credible intervals.* ***Abbreviations:*** *DAB+TRAM, combination dabrafenib and trametinib; IPI, ipilimumab; NIVO, nivolumab; PEM, pembrolizumab.*
